# Elucidation of salicylate attachment in celesticetin biosynthesis opens the door to create a library of more efficient hybrid lincosamide antibiotics†
†Electronic supplementary information (ESI) available. See DOI: 10.1039/c6sc04235j



**DOI:** 10.1039/c6sc04235j

**Published:** 2017-03-23

**Authors:** S. Kadlcik, Z. Kamenik, D. Vasek, M. Nedved, J. Janata

**Affiliations:** a Institute of Microbiology , Czech Academy of Sciences , BIOCEV , Prumyslova 595 , 252 50 Vestec , Czech Republic . Email: kamenik@biomed.cas.cz

## Abstract

Lincosamides, which are produced by streptomycetes, compose a small but clinically important class of antibiotics. The recent elucidation of the condensation and post-condensation biosynthetic steps of the lincosamides lincomycin and celesticetin revealed several unexpected reaction mechanisms. Here, we prepared recombinant proteins involved in the celesticetin biosynthetic pathway and used them for *in vitro* assays that were monitored by LC-MS. Our results elucidate the last biosynthetic step of celesticetin: the attachment of salicylic acid is catalyzed by the Ccb2 acyl-CoA ligase and the Ccb1 acyltransferase. Ccb1 belongs to the WS/DGAT protein family and, in contrast to the characterized members of the family, has unusual substrate specificity. To the best of our knowledge, Ccb1 is the first protein in this family that transfers a benzoyl derivative-CoA conjugate and is the first WS/DGAT protein involved in the biosynthesis of secondary metabolites. Furthermore, we exploited the relaxed substrate specificities of Ccb1 and Ccb2, as well as three additional upstream post-condensation biosynthetic proteins in the celesticetin pathway, and combined the lincomycin and the celesticetin biosynthetic pathways *in vitro*. In this way, we prepared a library of 150 novel hybrid lincosamides, including two unnatural chimeras of lincomycin and celesticetin, which were shown to have antibacterial properties more pronounced than clinically used lincomycin. These achievements may be considered a case study in applying knowledge about biosynthetic machinery to assemble a large number of compounds from originally a small group of natural products without the need for chemical synthesis.

## Abstract 2

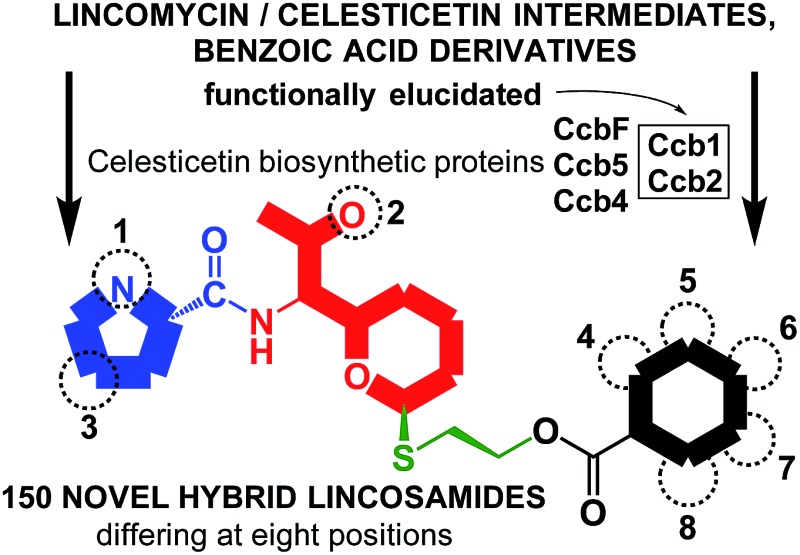
Combinatorial biosynthesis for more efficient antibiotics: 150 novel lincosamides prepared by combining lincomycin and celesticetin biosynthetic pathways.

## Introduction

**Fig. 1:**
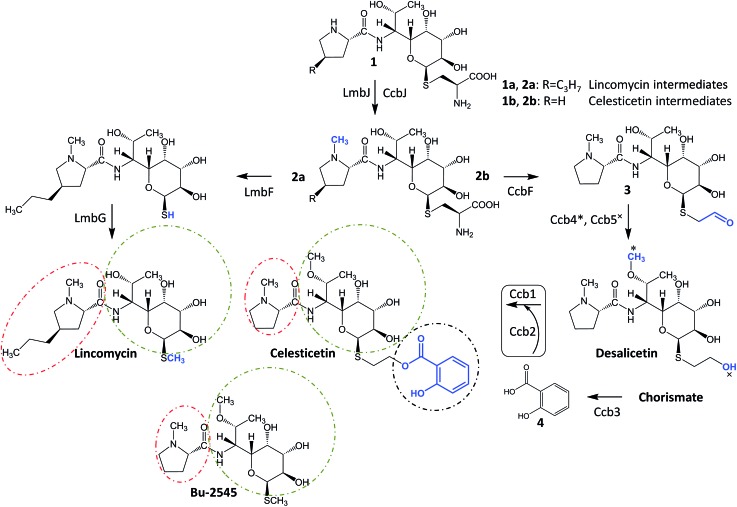
Natural lincosamides lincomycin, celesticetin and Bu-2545, and the terminal biosynthetic steps of lincomycin and celesticetin. The homologous proteins LmbF and CcbF process the mycothiol-derived cysteinyl moiety in different ways, leading to different ornamentation of the sulfur atom; the activation and attachment of salicylate to desalicetin (in the rectangle) is elucidated in this paper; final lincosamides have their moieties highlighted in ovals (amino acid – red, saccharide – green, organic acid – black); successively depicted biosynthetic steps are colored blue; Lmb: lincomycin biosynthetic proteins, Ccb: celesticetin biosynthetic proteins.

Lincosamides are antibacterial secondary metabolites produced by several *Streptomyces* species. Despite the small number of known natural lincosamides (limited to only three main compounds – lincomycin, celesticetin and Bu-2545, Fig. 1), lincomycin and its more efficient semisynthetic derivative, clindamycin, are clinically important antibiotics that are frequently used against Gram-positive bacteria. Additionally, clindamycin and several other derivatives have significant antiplasmodial effects.^1,2^ Thus, the development and application of an efficient combinatorial approach to prepare a large number of new lincosamide compounds would be an important prerequisite for the high-throughput search for more efficient antibacterial agents or even antimalarial drugs. Lincosamides are composed of an amino acid^3–6^ and an octose amino saccharide^7,8^ connected *via* an amide bond. The formation of the amide bond is catalyzed by an unusual condensation system that is coupled to the metabolism of two low-molecular-weight thiols: ergothioneine and mycothiol.^9,10^ These thiols are known for their roles in detoxification rather than biosynthesis; however, both are required for condensation and post-condensation reactions. Furthermore, the step-by-step elimination of mycothiol from the lincosamide intermediate during post-condensation maturation provides an opportunity to diversify the pathway towards lincomycin bearing a methylsulfhydryl group and celesticetin bearing a salicylate moiety connected *via* a two-carbon chain.^11^ This elimination step is specifically facilitated by the homologous pyridoxal-5′-phosphate-dependent proteins LmbF and CcbF during the biosynthesis of lincomycin and celesticetin, respectively, and these proteins process the mycothiol-derived cysteine residue present in intermediates **1** and **2** in different ways (Fig. 1). LmbF catalyzes β-elimination to form a sulfhydryl group,^11^ which is subsequently methylated by LmbG.^12,13^ By contrast, CcbF catalyzes decarboxylation-coupled oxidative deamination to preserve a two-carbon aldehyde attached at the sulfur atom, affording **3**.^11–13^ Following the reduction of the aldehyde to a hydroxyl group by the Ccb5 oxidoreductase,^12,13^ the intermediate, desalicetin, should be ready for salicylic acid (**4**) attachment by an unknown biosynthetic protein(s).

In this paper, we demonstrate that the final celesticetin biosynthetic step, *i.e.*, the condensation of desalicetin with salicylic acid to form celesticetin, is catalyzed by the cooperation of the Ccb2 salicylyl-CoA ligase and the Ccb1 acyltransferase. Furthermore, we show that both Ccb2 and Ccb1 exhibit broad substrate specificity, giving them the ability to attach a number of benzoic acid derivatives. In addition, we exploited the relaxed substrate specificities of CcbF, Ccb5 and a celesticetin-specific *O*-methyltransferase, Ccb4, to create a large library of 150 new hybrid, unnatural lincosamides using *in vitro* combinatorial biosynthesis.

## Results and discussion

### Attachment of salicylic acid is catalyzed by Ccb1 and Ccb2

We identified Ccb2 (548 amino acid residues, accession number ADB92576.1) and Ccb1 (437 amino acid residues, accession number ADB92559.1), which are encoded within the celesticetin biosynthetic gene cluster,^10^ as candidate proteins for the attachment of salicylic acid to desalicetin. Ccb2 shows sequence similarity to 2,3-dihydroxybenzoate- and salicylate-AMP ligases, which belong to the class I superfamily of adenylate-forming domains and are predicted to activate salicylic acid by adenylation. In the subsequent reaction step, acyl-AMP-ligases transfer salicylate from AMP to either a carrier protein, as would be typical of canonical non-ribosomal peptide synthetase systems,^14^ or to coenzyme A (CoA), as has been revealed in other systems.^15,16^ Since no suitable carrier protein is encoded within the celesticetin biosynthetic gene cluster, we presumed the utilization of CoA. Analysis of Ccb1 using BLASTp revealed that its N-terminal region is similar to those of the acyltransferases of the WS/DGAT family. These enzymes catalyze the final step in the biosynthesis of lipophilic storage compounds, triacylglycerols and wax esters.^17^ WS/DGAT-family proteins, including Ccb1, possess the highly conserved HHxxxDG motif.^18^ The HxxxxD motif is also present in several other enzyme families, including a broad spectrum of acyltransferases and non-ribosomal peptide synthetases.^17^ The catalytically active histidine in these enzymes initiates the deprotonation of a hydroxyl or amine group to enable a nucleophilic attack on the acyl donor,^19–23^ thus allowing the general reaction of these enzymes: the transfer of thioester-activated acyls to a hydroxyl or amine acceptor and the formation of an ester or amide bond. A BLASTp search found that the similarity between Ccb1 and its closest homolog, the putative WS/DGAT acyltransferase from *Mycobacterium obuense*, is very low (23.4% sequence identity). Sequence identities compared to characterized representatives of the WS/DGAT acyltransferases are 15.5% (AtfA), 20.0% (Tgs1), 19.2% (Atf1), and 21.1% (Sco1280); for the alignment of Ccb1 with characterized WS/DGAT proteins, see Fig. S1 in ESI.† Despite the low similarity to known proteins, we hypothesized that Ccb1 transfers salicylate from CoA to desalicetin.

We heterologously produced and purified Ccb2 and Ccb1 from *Escherichia coli*, and the purity and integrity of the recombinant proteins were assessed by SDS-PAGE (Fig. S2 in ESI†). Furthermore, we used gel filtration and blue native electrophoresis to determine that Ccb1 is monomeric in its native form (Fig. S3 in ESI†). By contrast, the characterized Ccb1 homolog from *Acinetobacter baylyi*, AtfA (458 amino acid residues), is a 94 kDa homodimer.^24^ We tested the Ccb2 and Ccb1 proteins *in vitro* with their expected substrates, *i.e.*, salicylic acid for Ccb2 and salicylyl-CoA conjugate and desalicetin or *O*-demethyldesalicetin for Ccb1. The two latter Ccb1 substrates were prepared by the alkaline hydrolysis of celesticetin and *O*-demethylcelesticetin and were purified by HPLC prior to their use as substrates.

**Fig. 2:**
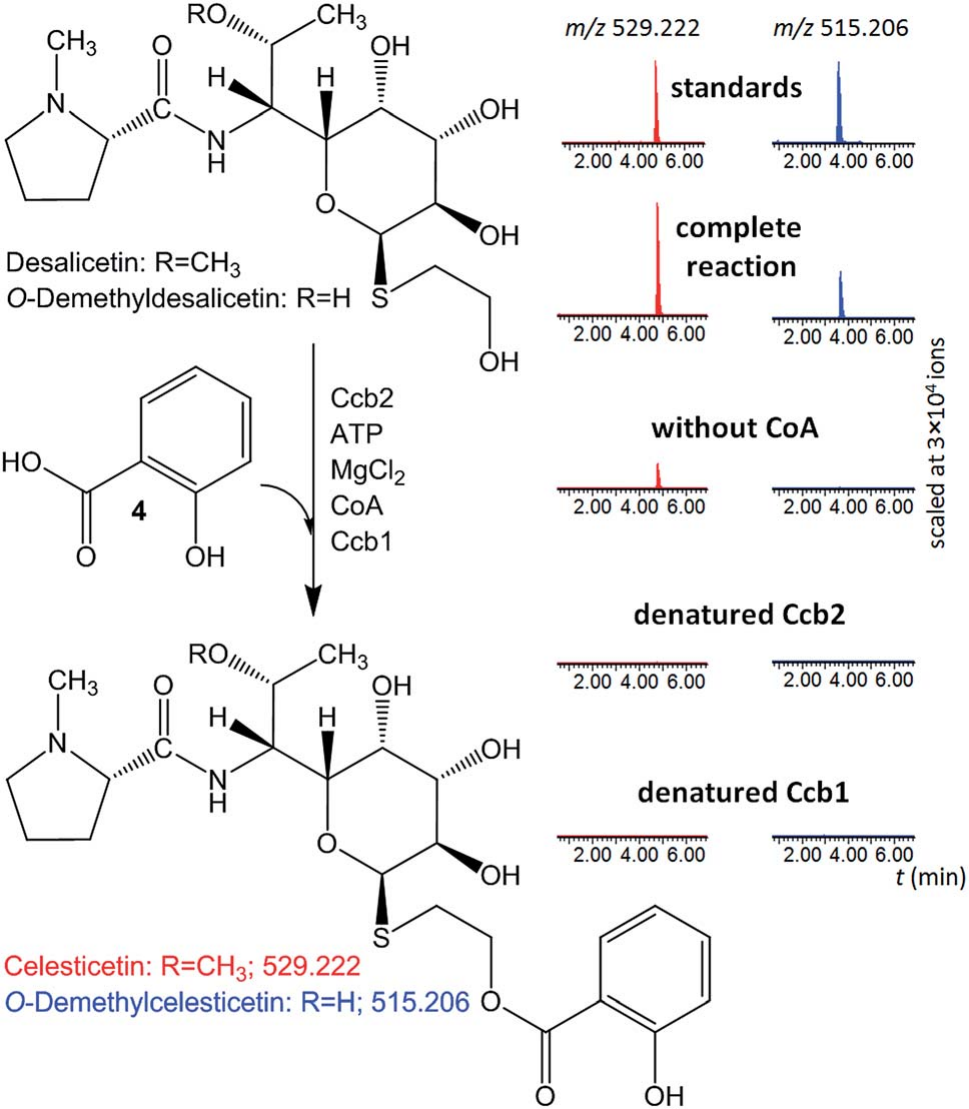
*In vitro* assays of salicylic acid (**4**) attachment to desalicetin or *O*-demethyldesalicetin catalyzed by Ccb2 and Ccb1; ion-extracted LC-MS chromatograms for the expected reaction products.

The ability of Ccb2 to adenylate salicylic acid was confirmed by the detection of an ion corresponding to a salicylyl-AMP conjugate using LC-MS (Fig. S4 in ESI†) and by a salicylic-acid-dependent exchange of radioactivity from [^32^P]-labeled PPi into ATP (data not shown). The subsequent Ccb2-catalyzed transfer of salicylic acid to CoA was also monitored by LC-MS (Fig. S4 in ESI†). We further included Ccb1 and desalicetin or *O*-demethyldesalicetin in the reaction. LC-MS analysis showed that salicylic acid (**4**) was attached to desalicetin or *O*-demethyldesalicetin, and the celesticetin or *O*-demethylcelesticetin product was detected (Fig. 2).

Both Ccb2 and Ccb1 were indispensable for this turnover because a reaction in which either protein had been heat-denatured yielded no product. When CoA was omitted, no *O*-demethylcelesticetin was detected as a product in the reaction with *O*-demethyldesalicetin. Interestingly, under analogous conditions, celesticetin was detected as a product in the reaction with desalicetin, but in lower amounts than in the reaction including CoA. This result is probably due to residual CoA, which may have co-purified with Ccb1 or Ccb2.

### Substrate specificity of Ccb1 and Ccb2

**Fig. 3:**
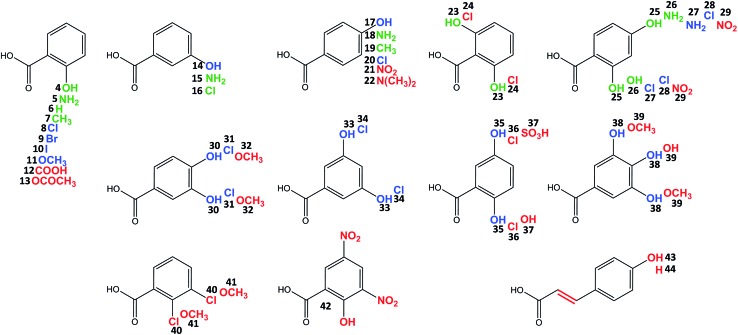
Benzoic acid derivatives tested for incorporation into lincosamides. Salicylic acid (**4**) is natural substrate; green: incorporation both *in vivo* and *in vitro*, blue: incorporation only *in vitro*, red: no incorporation.

Celesticetin derivatives with anthranilic or 4-amino-2-hydroxybenzoic acids incorporated *in vivo* into the structure instead of salicylic acid were described previously.^25,26^ These reports encouraged us to explore the substrate specificities of Ccb2 and Ccb1 in more detail. Specifically, we tested the ability of Ccb2 and Ccb1 to activate and attach the 40 benzoic acid derivatives **5–44** listed in Fig. 3. First, we supplemented an agar culture medium with a benzoic acid derivative and inoculated it with the celesticetin-producing strain *Streptomyces caelestis*. Although the biosynthesis of natural celesticetin remained unaffected by the fed compounds, the incorporation of benzoic acid derivatives was observed only in the case of acids **5**, **15**, and **18**, which bear an amino group; benzoic acid (**6**); acids **7** and **19**, which bear a methyl group; 3-chlorobenzoic acid (**16**); two dihydroxy derivatives **23** and **25**; and 4-amino-2-hydroxybenzoic acid (**26**) (green in Fig. 3). We repeatedly failed to incorporate any of the other derivatives into the structure *in vivo*, which we found disappointing, particularly for the hydroxybenzoic acids. Therefore, we tested all 40 benzoic acid derivatives *in vitro* with Ccb2 and monitored the benzoyl derivative-AMP conjugates. We detected products (LC-MS data are available in Fig. S5 in ESI†) for those derivatives with the successful *in vivo* feeding experiment, as well as for 15 other acids: **8–11**, **14**, **17**, **20**, **27**, **28**, **30**, **31**, **33–35**, **38**. We used the benzoic acid derivatives that were activated by Ccb2 for the *in vitro* production of celesticetin derivatives by Ccb1. We detected expected products for all of them when desalicetin was used as the substrate (blue in Fig. 3) and for all except **11** and **38** when *O*-demethyldesalicetin was used as the substrate (for LC-MS data see Fig. S6–S31 in ESI†). Ccb2 failed to activate **12** with an additional carboxyl group, as well as derivatives **21**, **29**, and **42**, containing (a) nitro group(s); derivatives with bulky substituents such as acetyl (**13**); at least two methoxy groups (**32**, **39**, **41**); a sulfo group (**37**); a substituted amino group (**22**); and acids **43** and **44** with an additional two carbon chain. Interestingly, acids with chlorines in positions 2,3-; 2,5- and 2,6- (**40**, **36**, **24**) were not activated, while acids with chlorines in positions 2,4-; 3,4- and 3,5- (**28**, **31**, **34**) were successfully activated and incorporated. By contrast, all tested combinations of dihydroxy derivatives, *i.e.*, in positions 2,4-; 2,5-; 2,6-; 3,4- and 3,5- (**25**, **35**, **23**, **30**, **33**) were processed by both Ccb2 and Ccb1 and converted to the products.

### Creation of hybrid lincosamides

**Fig. 4:**
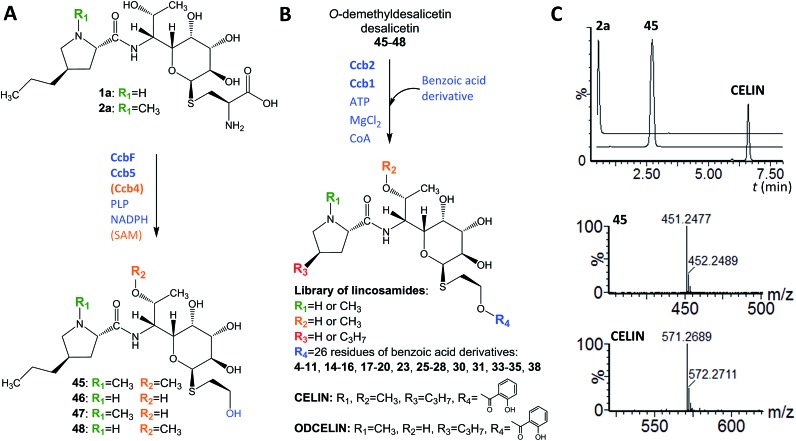
Library of hybrid lincosamides. (A) Lincomycin intermediates **1a** and **2a** processed by celesticetin biosynthetic proteins, affording intermediates **45–48**; (B) attachment of benzoic acid derivatives **4–44** to *O*-demethyldesalicetin, desalicetin, and intermediates **45–48**, resulting in a library of hybrid lincosamides including 150 novel compounds. (C) Ion-extracted chromatograms of intermediates **2a** (*m*/*z* 480.2380), **45** (*m*/*z* 451.2487) and hybrid lincosamide CELIN (*m*/*z* 571.2690), and MS spectra of the two latter compounds.

In looking at the biosynthetic steps preceding salicylic acid incorporation (Fig. 1), one could ask if it is possible to process celesticetin intermediate **2b** through the lincomycin biosynthetic route and, conversely, lincomycin intermediate **2a** through the celesticetin route. The *in vitro* processing of celesticetin intermediate **2b** by LmbF, LmbG, and Ccb4 was demonstrated previously^12,13^ and resulted in compound Bu-2545 (Fig. 1), a naturally occurring lincosamide produced by *Streptomyces* strain no. H230-5.^27^ Herein, we present the successful *in vitro* processing of lincomycin intermediate **2a** by the celesticetin biosynthetic proteins CcbF, Ccb5, and Ccb4 into the intermediate **45** and its subsequent conversion by Ccb2 and Ccb1, resulting in a novel hybrid lincosamide, CELIN, which combines the 4-propyl side chain of lincomycin and an *O*-methyl group, a two-carbon chain and a salicylate from celesticetin (see Fig. 4 for the scheme and LC-MS data). Inspired by these experiments, we created a library of unnatural lincosamides prepared *via* intermediates **45–48**. The library was diversified as follows: (1) the use of **2a** and its *N*-demethylated derivative **1a** – specifies R_1_ in Fig. 4 (**1a** and **2a** were purified from a deletion mutant of the lincomycin-producing strain *Streptomyces lincolnensis* and were previously structurally elucidated by MS and NMR^11^); (2) the inclusion or omission of the Ccb4 methyltransferase – specifies R_2_ in Fig. 4; and (3) the incorporation of benzoic acid derivatives other than salicylic acid – specifies R_4_ in Fig. 4. Including the aforementioned incorporation of benzoic acid derivatives into desalicetin and *O*-demethyldesalicetin, we prepared 152 lincosamides, of which 150 are novel (summarized in Fig. 4, for LC-MS data including collision-induced dissociation fragmentation MS spectra, see Fig. S6–S31 in ESI†).

### Assessment of antibacterial properties

**Fig. 5:**
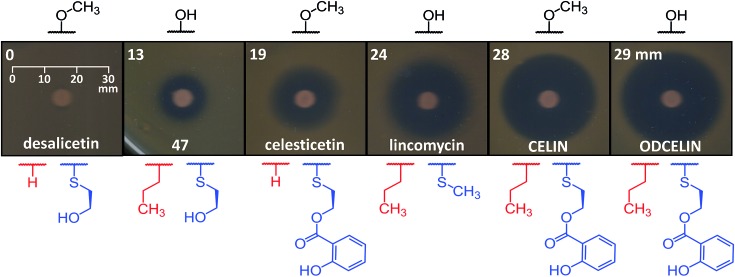
Disc diffusion bioassay with Gram-positive strain *K. rhizophila*. Structural differences between the compounds include *O*-methylation at the saccharide moiety (black), the side chain at the amino acid moiety (red), and ornamentation of the sulfur atom (blue); diameter of the inhibition zones is stated in the upper left corner.

To evaluate the antibacterial potential of the library of hybrid lincosamides, we conducted bioassays with the lincomycin-sensitive Gram-positive strain *Kocuria rhizophila* and tested six compounds: lincomycin and celesticetin as natural products; CELIN and its *O*-demethylated derivative, ODCELIN, as representatives of the library of hybrid lincosamides; and finally desalicetin and **47**, *i.e.*, salicylate-free intermediates derived from celesticetin and ODCELIN, respectively. We performed a disc diffusion test on solid media (Fig. 5) and for four most potent antimicrobials, we determined minimal inhibition concentrations in liquid media with the results as follows: celesticetin 1600 nM, lincomycin 400 nM, CELIN 100 nM, ODCELIN 100 nM. Both types of bioassays were in accordance and gave us brief insight into the structure–bioactivity relationship of the tested compounds. First, *O*-methylation appears to have only limited effect on antibacterial activity (compare CELIN *vs.* ODCELIN). Second, 4-propyl chain at the proline moiety significantly enhances the antibacterial activity (compare celesticetin *vs.* CELIN), which agrees with previous reports showing that the length of this side chain significantly affects the antibacterial activity.^1^ Third, compounds bearing salicylate showed considerably more antibacterial effect compared to compounds bearing a free hydroxyl group (compare desalicetin *vs.* celesticetin and **47** *vs.* ODCELIN). What is important, the significance of salicylate moiety applies also to compounds differing in the *S*-functionalization entirely (compare clinically used lincomycin *vs.* ODCELIN). This report thus for the first time shows, that not only the propyl chain of the proline moiety, but also salicylate moiety is important for antimicrobial activity of lincosamides.

## Conclusions

The elucidation of the final step in celesticetin biosynthesis revealed that Ccb1 accepts all 26 tested substrates that were activated by Ccb2. Therefore, it is Ccb2 that controls whether the benzoic acid derivative will be selected for incorporation into the lincosamide, while the role of Ccb1 in benzoic acid derivative selection seems limited. However, we assume that Ccb1 determines the substrate to which the benzoic acid derivative will be attached and that this substrate is restricted to lincosamides. This prediction also explains the low sequence homology between Ccb1 and all other acyltransferases of the WS/DGAT family, which typically synthesize triacylglycerols and wax esters by the esterification of CoA-activated fatty acids and diacylglycerols or long-chain fatty alcohols, respectively. It is noteworthy that Ccb1 is the first characterized WS/DGAT protein involved not in triacylglycerol or wax ester synthesis but in a specific condensation reaction in secondary metabolism; it is also the first WS/DGAT protein with specificity for benzoyl derivative-CoA compounds.

The bioassay experiments suggested that the library of 150 new hybrid lincosamides has a pharmaceutical potential and that the unnatural combination of natural substrates and biosynthetic proteins resulted in more effective antibiotics. Importantly, the library has great potential to expand further. Given that Ccb2 activates benzoic acid derivatives with CoA and not a specific carrier protein, one can imagine using CoA-ligases from different biosynthetic machineries that activate compounds with structural motifs other than benzoic acid derivatives. The resulting acyl-CoA conjugates of these compounds would likely be accepted by Ccb1 for attachment to a lincosamide.

Regarding the preparation of hybrid lincosamides, there is another interesting condensation system involved in lincosamide biosynthesis. In addition to the Ccb2/Ccb1 system reported here, which connects organic acid and saccharide units, another previously described system connects amino acid and saccharide units in a different, more complex process.^9,10,28^ In both systems, there is an acyl-activating protein that is responsible for selecting the acid unit. Similar to the broad substrate specificity of Ccb2, the amino acid-activating protein from lincomycin biosynthesis, LmbC, not only accepts its main natural substrate, 4-propyl-l-proline but also readily activates 4-butyl-l-proline and 4-pentyl-l-proline.^28^ This feature of LmbC enabled the production of lincosamides with longer alkyl side chains at the amino acid unit using mutasynthesis.^29^ The combination of lincomycin and celesticetin biosynthesis and the exploitation of the broad substrate specificities of the ‘selecting enzymes’ Ccb2 and LmbC could multiply the number of potentially accessible lincosamides.

In conclusion, this study demonstrates a way to diversify natural products using combinatorial biosynthesis, resulting in the unprecedented enlargement of a small but important class of biologically active compounds with no need for chemical synthesis.
