# Supplementary material for: Elucidation of salicylate attachment in celesticetin biosynthesis opens the door to create a library of more efficient hybrid lincosamide antibiotics
Source: Chem Sci. 2017 Mar 23;8(5):3349–55. doi: 10.1039/c6sc04235j (PMC5416915; doi:10.1039/c6sc04235j)
Supplement: Supplementary file 1 [file SC-008-C6SC04235J-s001.pdf]

Electronic Supplementary Material (ESI) for Chemical Science.
This journal is © The Royal Society of Chemistry 2017

Elucidation of salicylate attachment in celesticetin biosynthesis opens the door to create a library of more efficient hybrid lincosamide antibiotics

S. Kadlcik, Z. Kamenik*, D. Vasek, M. Nedved, J. Janata

Institute of Microbiology, Videnska 1083 142 20, Prague 4, Academy of Sciences of the Czech Republic

*kamenik@biomed.cas.cz

Electronic Supplementary Information

Supplementary material and methods

Chemicals

Lincomycin (863 units per mg), acetylsalicylic (**13**, >98%), 3-aminobenzoic (**15**, 98%), 4-amino-2-chlorobenzoic (**22**, 97%), 4-amino-2-hydroxybenzoic (**26**, 99%), benzoic (**6**, >97%), 2-chlorobenzoic (**8**, 98%), *trans*-cinnamic (**44**, 99%), 4-coumaric (**43**, 98%), 2,3-dichlorobenzoic (**40**, 97%), 2,5-dichlorobenzoic (**36**, 97%), 3,4-dichlorobenzoic (**31**, 99%), 3,4-dihydroxybenzoic (**30**, 97%), 3,4-dimethoxybenzoic (**32**, 99%), 2,4-dinitrobenzoic (**29**, 96%), 3-hydroxybenzoic (**14**, 99%), 4-hydroxybenzoic (**17**, 99%), 3,4,5-trihydroxybenzoic (**38**, gallic, 99%), 2-methylbenzoic (**7**, 95%), 4-methylbenzoic (**19**, 98%), 2-hydroxybenzoic (**4**, salicylic, >97%), 4-hydroxy-3,5-dimethoxybenzoic (**39**, syringic, >95%) acids were obtained from Sigma-Aldrich (Germany); 4-aminobenzoic (**18**, 99%), 2-bromobenzoic (**9**, >98%), 3,5-dichlorobenzoic (**34**, >98%), 2,4-dihydroxybenzoic (**25**, >95%), 2,5-dihydroxybenzoic (**35**, >98%), 3,5-dihydroxybenzoic (**33**, >90%), 2-iodobenzoic (**10**, 95%), and 4-nitrobenzoic (**21**, >95%) acids were obtained from Fluka; 3-chlorobenzoic (**16**, >98%), 4-chlorobenzoic (**20**, >98%), 2,4-dichlorobenzoic (**28**, 98%), 2,6-dichlorobenzoic (**24**, >98%), 2,6-dihydroxybenzoic (**23**, >97%) acids were obtained from Merck-Schuchardt (Germany); 2-aminobenzoic (**5**, anthranilic, 99%), 2-hydroxy-3,5-dinitrobenzoic (**42**, >97%), phthalic (**12**, >95%), and 2-hydroxy-5-sulfobenzoic (**37**, p.a.) acids were obtained from Lachema (Czech Republic); 2,3-dimethoxybenzoic acid (**41**, >97%) was obtained from Loba-Chemie (Austria), 2-methoxybenzoic (**11**) and 4-(dimethylamino)benzoic acids (**22**) were obtained from Assoc. Prof. Jindrich Jindrich, Faculty of Sciences, Charles University in Prague. Adenosine 5´-triphosphate (ATP, >99%), *S*-adenosyl-methionine (SAM, 80%), ammonium hydroxide (28-30% solution), coenzyme A disodium salt (CoA, >96%), β-nicotinamide adenine dinucleotide phosphate reduced (NADPH, 96%), pyridoxal-5´-phosphate (PLP, >98%) were obtained from Sigma-Aldrich (Germany), formic acid (98-100%) from Merck (Germany), acetonitrile and methanol (both LC-MS grade) from Biosolve (Netherlands).

Bacterial strains

The *Streptomyces lincolnensis* Δ*lmbIH* mutant strain was derived from lincomycin producer *S. lincolnensis* ATCC 25466 as decribed previously.[1] The strain was used for production of lincomycin intermediates **1a** and **2a**. Celesticetin producer *Streptomyces caelestis* ATCC 15084 was used for production of celesticetin and *O*-demethylcelesticetin and as a source of genomic DNA for gene

amplification. Routine DNA manipulations were performed in *E. coli* XL1 Blue MR[b] (Stratagene). The heterologous overproduction of *S. caelestis* proteins was performed in *E. coli* BL21(DE3) (Novagen).

Cultivation of streptomycete strains in liquid media

The seed culture of *S. lincolnensis* Δ*lmbIH* or *S. caelestis* was prepared by inoculation of spores from MS plates into 50 mL of the YEME medium[2] without sucrose and incubated in 500 mL flat-bottom boiling flasks at 28 °C. Two mL of 24 h seed culture were inoculated into 40 mL of AVM[3] (*S. lincolnensis*) or GYM[4] medium (*S. caelestis*) and incubated in 500 mL flat-bottom boiling flasks at 28 °C for 120 h. The cells were centrifuged at 5000 g at 20 °C for 15 min and the supernatant was used for LC-MS analyses or purification of metabolites of interest.

In vivo incorporation experiments with benzoic acid derivatives

Plates with 10 mL of agar GYM medium supplemented with 2 mM benzoic acid derivative were inoculated with spores of *S. caelestis*. The plates were incubated at 28 °C for 8 days. Three discs 5 mm in diameter were cut from the agar medium, inserted into 500 µL methanol, vortexed 1 min and incubated in a sonication bath for 15 min. Then, 200 µL of the methanolic extract was removed, evaporated to dryness, reconstituted in 50 µL methanol:water 50:50 (v/v) and analyzed by LC-MS.

Preparation of desalicetin and *O*-demethydesalicetin from celesticetin and *O*-demethylcelesticetin and purification of lincomycin intermediates 1a and 2a, lincosamides CELIN and ODCELIN, and intermediate 47

Celesticetin and *O*-demethylcelesticetin were purified from the culture broth of *S. caelestis*. Intermediates **1a** and **2a** were purified from the culture broth of the *S. lincolnensis* Δ*lmbIH* mutant strain. The compounds were purified as follows. An Oasis HLB 6cc 200 mg cartridge (hydrophilic-lipophilic balanced sorbent, Waters, USA) was conditioned with 5 mL methanol, equilibrated with 5 mL water, and then 50 mL cultivation broth was loaded. Subsequently, the cartridge was washed with 5 mL water and absorbed substances were eluted with 15 mL methanol. The eluent was evaporated to dryness, reconstituted in 2 mL methanol, and the extracts were injected into the HPLC apparatus equipped with flow controller 600, autosampler 717, and UV detector 2487 operating at 194 nm (Waters, USA). Data were processed with Empower 2 software (Waters, USA). The analytes were separated on the Luna C18 chromatographic column (250 × 15 mm I.D., particle size 5 µm, Phenomenex, USA) with the two component mobile phase, A and B, consisting of 0.1% formic acid and methanol, respectively. The analyses were performed under a linear gradient program (min/%B) 0/5, 31/27.5 followed by a 9-min column clean-up (100% B) and a 9-min equilibration (5% B), at a flow rate of 3 mL min^{-1}. The fractions containing celesticetin and *O*-demethylcelesticetin were treated with 0.1mM NaOH overnight, resulting in complete conversion of the compounds into desalicetin and *O*-demethydesalicetin, respectively. Intermediates **1a**, **2a** as well as desalicetin and *O*-demethydesalicetin were subjected to another HPLC purification using the XTerra Prep RP18 column (150 × 7.8 mm I.D., particle size 5.0 µm, Waters, USA). The analytes were eluted using the isocratic program with 1 mM ammonium formate (pH 9.0):acetonitrile (90:10 v/v) as mobile phase. Compounds CELIN, ODCELIN and **47** were purified from the scaled-up in vitro reactions - see below the paragraph Preparation of hybrid lincosamides from intermediates **1a** and **2a**; point (1) applies for compound **47** and points (1) and (2) apply for CELIN and ODCELIN. Proteins in the in vitro assays were precipitated by 98% formic acid (30 µL per 1 mL sample) and the supernatant was subjected to

purification using Oases HLB cartridges and HPLC as described above for purification of intermediates **1a** and **2a** with the following exceptions. Two component mobile phase, A and B, consisting of 0.1% formic acid and acetonitrile, respectively, was used. The analyses were performed under a linear gradient program (min/%B) 0/30, 40/65 followed by a 9-min column clean-up (100% B) and a 9-min equilibration (30% B). Single purification step by HPLC was considered as sufficient. The fractions containing the separated compounds were checked for purity (>95% - LC with UV detection at 220 nm; no significant cross-contamination).

Heterologous production and purification of CcbF, Ccb1, Ccb2, Ccb4, and Ccb5

The *ccbF, ccb1, ccb2, ccb4,* and *ccb5* genes were PCR-amplified from the genomic DNA of *S. caelestis* using the primer pairs listed in Table S1. The *ccbF* and *ccb2* genes were inserted into the pET42b vector (Novagen), and the *ccb1*, *ccb4*, and *ccb5* genes were inserted into the pET28b vector (Novagen). The resulting constructs were used to produce soluble C-terminally (CcbF and Ccb2) or N-terminally (Ccb1, Ccb4, and Ccb5) His-tagged fusion proteins in *E. coli* BL21 (DE3) with the GroES and GroEL chaperonins co-expressed. Overproduction was induced with 0.4 mM IPTG and was performed at 17 °C for 20 h. The cells were harvested by centrifugation (4200 rpm, 20 min, 4 °C), and cell-free extracts were prepared using ultrasonic homogenization in a binding buffer consisting of 20 mM TRIS pH 8, 100 mM NaCl, 10% glycerol, and 20 mM imidazole followed by centrifugation (9000 rpm, 25 min, 4 °C). The proteins were purified from the cell-free extracts using a 1 mL HiTrap^{TM} Ni^{2+} column (GE Healthcare). Ccb2 was eluted with binding buffer supplemented with 100 mM imidazole, Ccb4 and Ccb5 were eluted with binding buffer supplemented with 200 mM imidazole and CcbF and Ccb1 were eluted with binding buffer supplemented with 250 mM imidazole. Imidazole was eliminated from the purified proteins by buffer exchange using 30-kDa Amicon cartridges (Millipore) and binding buffer without imidazole. Ccb4 was stable for at least a week at 4 °C with no decrease in activity; CcbF, Ccb1, Ccb2, and Ccb5 were stable for at least a week at 4 °C or a month at -80 °C with no decrease in activity. Protein concentration was determined with a Nanodrop ND-1000 Spectrophotometer (Thermo Scientific, USA) at 280 nm using extinction coefficients calculated from the protein sequences (98 890 for CcbF; 91 440 for Ccb1, 70 360 for Ccb2, 28 420 for Ccb4, and 24 410 for Ccb5).

Amplified gene	Forward primer	Reverse primer
ccbF	CCGCATATGTCCGACTTAGCTGCCG	CCGCTCGAGGCGGGGCTGCCAGGCG
ccb1	CTGCATATGCATCTTGATCCAACCAC	ATAGAATTCTCATCGGTGGTCGTCGC
ccb2	AACCCCATATGAAGCGACGTGGCATGG	AACCCCTCGAGTAAGGTCATGAACTCCGCACG
ccb4	CTACATATGAAGACGCCCGGTACATC	CTAGAATTCTCAGCACGGAGTGGCCT
ccb5	ATAGCTAGCGCGACCGTCCCCGCC	CTGGAATTCTCATGAGTCCGCGCGCC

Table S1. Primers used for amplification of *S. caelestis* genes. Restriction sites used for insertion into the vector are underlined.

Gel filtration

The monomeric or oligomeric form of Ccb1 was determined by gel filtration with a Superose 12 HR 10/30 column (Pharmacia) in a buffer containing 20 mM Tris pH 8, 100 mM NaCl, 10% glycerol; the flow rate was 0.5 mL min^{-1}. BSA was used as a standard.

Blue native electrophoresis

This experiment was performed as described by Schägger et al.[5] in a linear acrylamide concentration gradient of 8-18%.

Bioinformatic tools

The BLASTX and BLASTP were used for prediction of functions of encoded proteins (http://blast.ncbi.nlm.nih.gov/Blast.cgi) in combination with Conserved Domains Database (http://www.ncbi.nlm.nih.gov/cdd). The amino acid sequences of the WS/DGAT proteins were retrieved from GenBank. Sequence identities of these proteins to Ccb1 were calculated in Geneious 5.5.9 (http://www.geneious.com, Kearse et al.[6]) based on pairwise alignments generated using MAFFT software version 7.304b at the CBRC web site (http://mafft.cbrc.jp/alignment/server, Katoh et al.[7]). The multiple sequence alignment of WS/DGAT proteins was generated using the MAFFT and visualized in the Geneious.

In vitro assays

Preparation of benzoyl derivative-AMP conjugates (assays with Ccb2): 2 µM Ccb2, 2 mM benzoic acid derivative, 4.5 mM ATP, 2 mM $MgCl_2$, 100 mM Tris pH7.5 assay was conducted to monitor the AMP-benzoic acid derivative conjugates. The reaction was supplemented with 2 mM coenzyme A to monitor the benzoyl derivative-CoA conjugates. The assays were incubated at 30 °C for 2 h.

Preparation of hybrid lincosamides from desalicetin or *O*-demethyldesalicetin (assays with Ccb2 and Ccb1): 2 µM Ccb2, 2 µM Ccb1, 200 µM desalicetin or *O*-demethyldesalicetin, 2 mM benzoic acid derivative, 4.5 mM ATP, 2 mM coenzyme A, 2 mM $MgCl_2$, 100 mM Tris pH 7.5. The assays were incubated at 30 °C for 2 h.

Preparation of hybrid lincosamides from intermediates 1a and 2a (assays with CcbF, Ccb5, Ccb4, Ccb2, Ccb1):

(1) 20 µM CcbF, 20 µM Ccb5, 200 µM **1a** or **2a**, 200 µM PLP, 1 mM NADPH, 100 mM Tris pH7.5; in the case of *O*-methyl derivatives preparation 20 µM Ccb4 and 4 mM SAM were additionally included in the reaction. The assays were incubated at 30 °C for 2 h.

(2) 2 µM Ccb2, 2 µM Ccb1, 2 mM benzoic acid derivative, 4.5 mM ATP, 2 mM coenzyme A, 2 mM $MgCl_2$, 100 mM Tris pH 7.5. The assays were incubated at 30 °C for 30 min.

Reactions (1) and (2) were mixed and incubated at 30 °C for additional 2 h.

Negative controls were performed with proteins inactivated by heat (90 °C, 15 min). Reactions were terminated by adding 1 µL formic acid per 50 µL reaction, centrifuged (3000 g, 5 min) and analysed by LC-MS.

Assay of antibacterial properties

The filtration paper discs (diameter, 5 mm) containing 5 nmol of the tested compound were transferred onto plates overlaid with the lincomycin-sensitive strain *Kocuria rhizophila* and the plates were cultivated for 20 h at 37 °C.

Minimal inhibition concentrations were determined as published previously.[8] Five µL of 0.5 McFarland suspension of *K. rhizophila* culture was inoculated into 1 mL LB medium supplemented with the tested compounds at the required concentration (tested concentration range: 25–1600 nM). The cultures were incubated for 24 h at 37 °C.

LC-MS analyses

Lincosamides and salicylyl-CoA conjugate. LC-MS analyses were performed on the Acquity UPLC system with LCT premier XE time-of-flight mass spectrometer (Waters, USA). Five µL of sample were loaded onto the Acquity UPLC BEH C18 LC column (50 mm × 2.1 mm I.D., particle size 1.7 µm, Waters) kept at 40 °C and eluted with a two-component mobile phase, A and B, consisting of 0.1% formic acid and acetonitrile, respectively. The analyses were performed under a linear gradient program (min/%B) 0/5, 1.5/5, 12.5/58 followed by a 1.5-min column clean-up (100% B) and 1.5-min equilibration (5% B), at the flow rate of 0.4 mL min^{-1}. The mass spectrometer operated in the "W" mode with capillary voltage set at +2800 V, cone voltage +40 V, desolvation gas temperature, 350 °C; ion source block temperature, 120 °C; cone gas flow, 50 Lh^{-1}; desolvation gas flow, 800 Lh^{-1}; scan time of 0.15 s; inter-scan delay of 0.01 s. The mass accuracy was kept below 5 ppm using lock spray technology with leucine enkephalin as the reference compound (2 ng μL^{-1}, 5 µL min^{-1}). Fragmentation by collision-induced dissociation (CID) was triggered by increasing aperture 1 at 50 V. Chromatograms were extracted for $[M+H]^+$ ions with the tolerance window of 0.05 Da. The data were processed by MassLynx V4.1 (Waters).

Benzoyl derivative-AMP conjugates. The analyses were performed as above described for lincosamides with the following exceptions. Acquity UPLC BEH Amide column (50 mm × 2.1 mm I.D., particle size 1.7 µm, Waters) kept at 30 °C was used for separation using a two-component mobile phase, A: acetonitrile and B: 50 mM ammonium acetate pH 5.8:acetonitrile 50:50 (v/v). The analyses were eluted in a linear gradient program (min/%B) 0/5, 2.5/5, 8/73.9 followed by a 1.5-min column clean-up (99% B) and 1.5-min equilibration (5% B).

Supplementary results

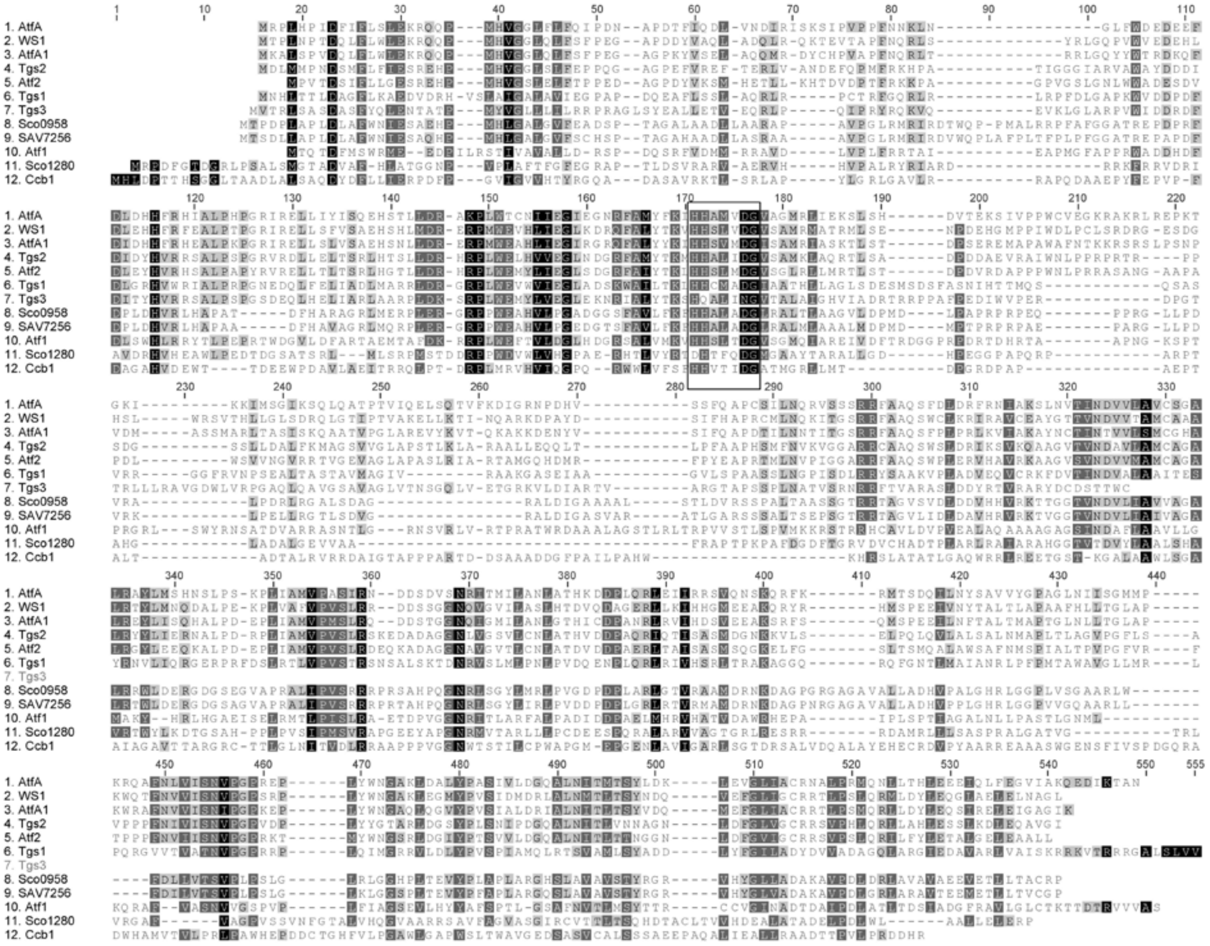

Fig. S1 Multiple sequence alignment of Ccb1 and characterized WS/DGAT enzymes. The alignment was generated using the MAFFT web tool and was visualized with Geneious software. Residues that are identical or similar are highlighted. The conserved HHxxxDG motif is in the rectangle.

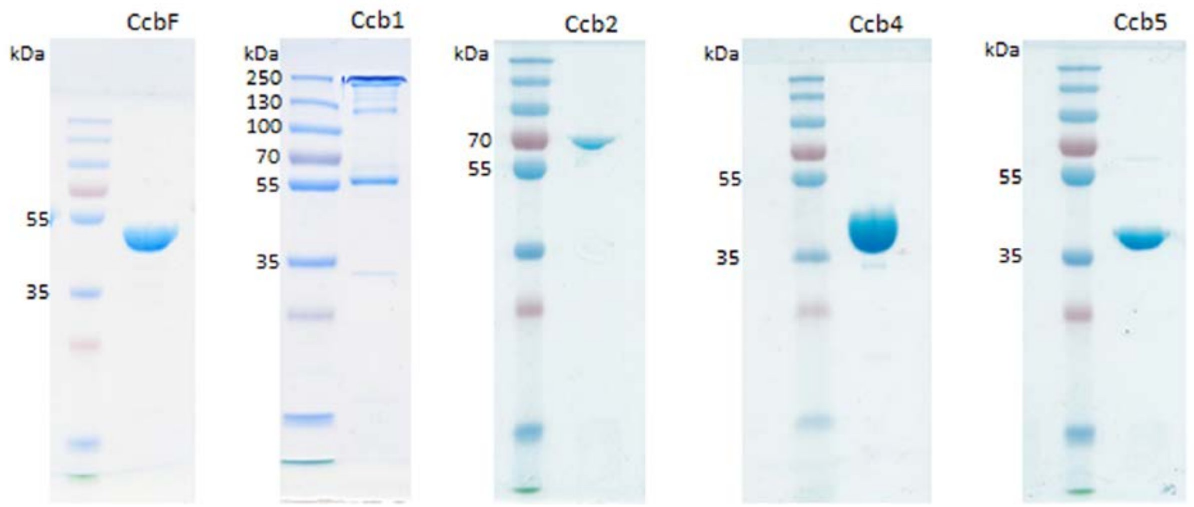

Fig. S2 SDS-PAGE analysis of purified recombinant CcbF, Ccb1, Ccb2, Ccb4, and Ccb5 proteins. The theoretical MWs are as follows: His_8-tagged CcbF, is 48.0 kDa; His_6-tagged Ccb1, 49.3 kDa; His_8-tagged Ccb2, 60.4 kDa; His_6-tagged Ccb4, 43.0 kDa; His_6-tagged Ccb5, 38.8 kDa; MW standard: PageRuler™ prestained protein ladder.

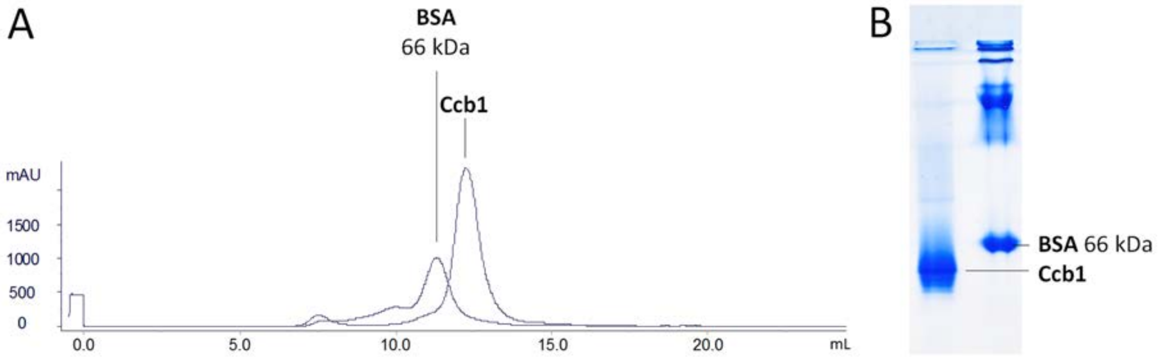

Fig. S3 Gel filtration profile (A) and blue native electrophoresis (B) of Ccb1. The theoretical MW for His_6-tagged Ccb1 in its monomeric form is 49.3 kDa. Both methods show that Ccb1 is a monomer in its native form.

Note for Figs. S2 and S3:

Ccb1 forms aggregates in SDS-PAGE, particularly at approximately 250 kDa. Gel filtration and blue native electrophoresis show that Ccb1 is a monomer under native conditions, with the results corresponding to the theoretical MW (49.3 kDa). These observations suggest that the aggregates observed on SDS-PAGE are formed as a consequence of the denaturation process.

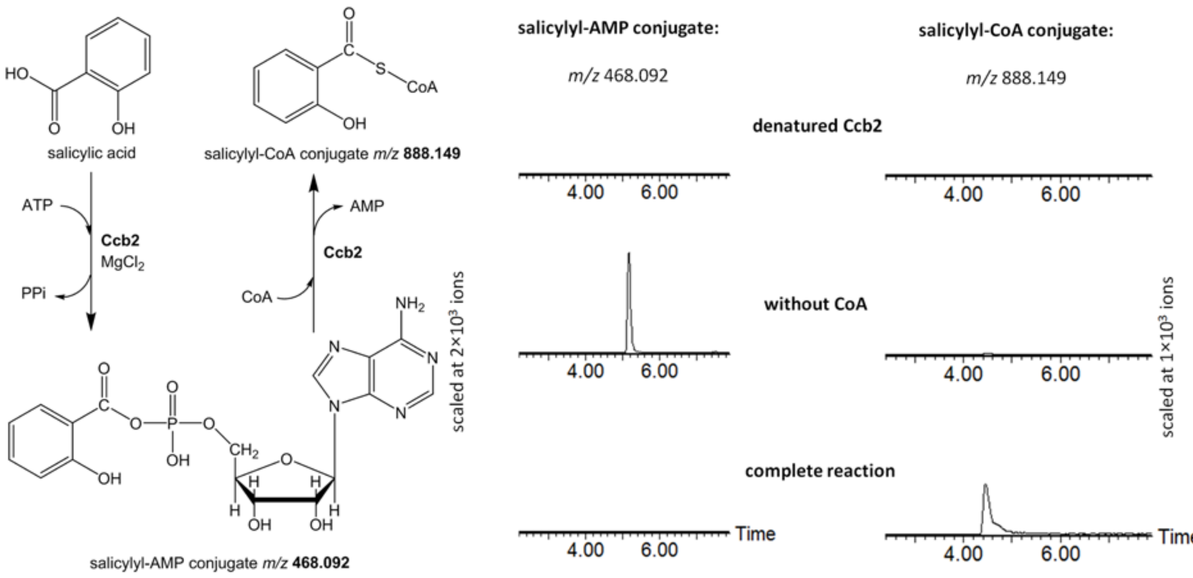

Fig. S4 In vitro assay with Ccb2. Activation of salicylic acid by adenylation and subsequent transfer to coenzyme A. Ion extracted LC-MS chromatograms for the expected products of the reaction.

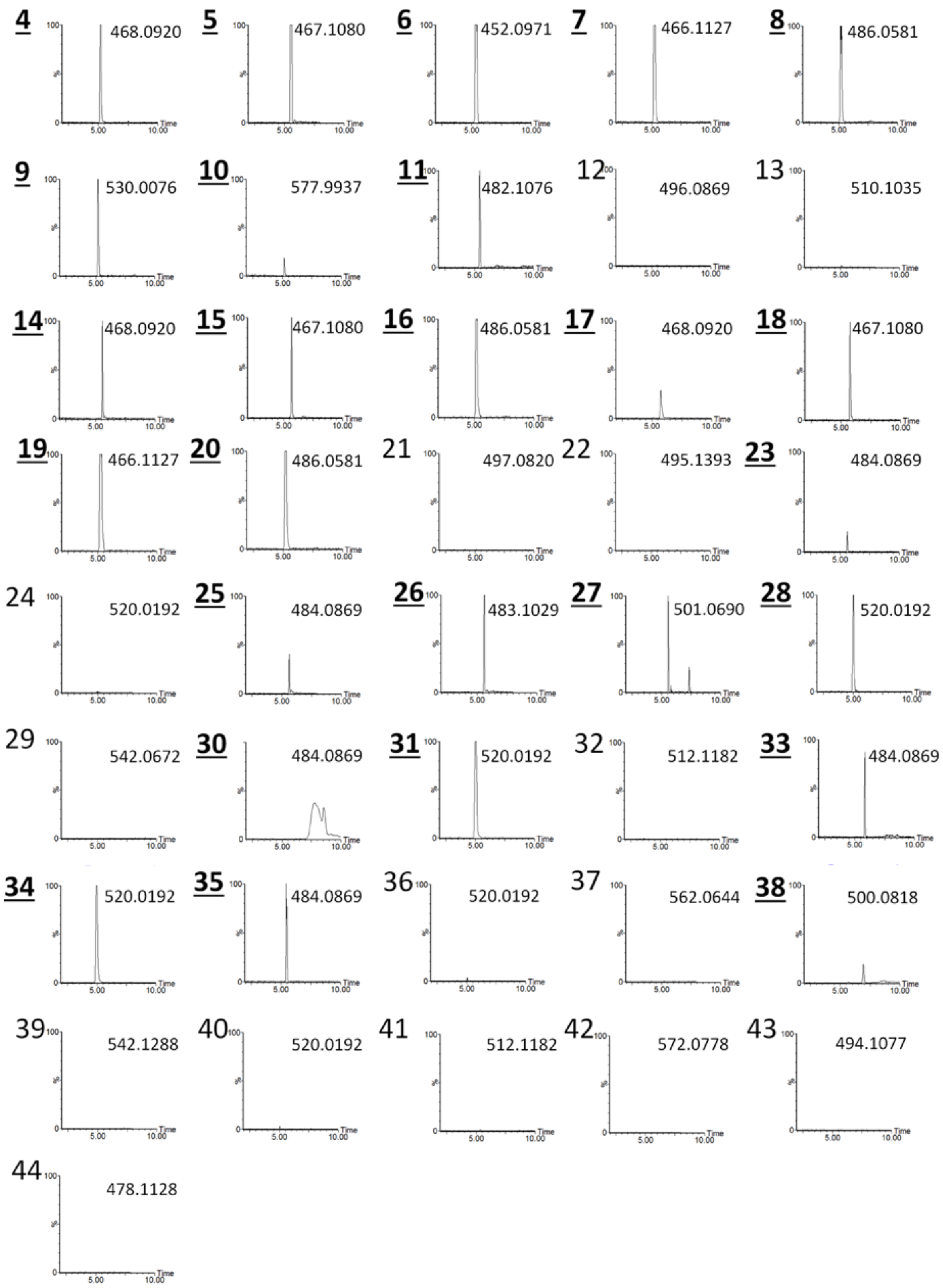

Fig. S5 Formation of benzoyl derivative-AMP conjugates catalyzed by Ccb2. Ion-extracted chromatograms (scaled at 10^3 ions, *m/z* stated in the chromatograms); the numbers correspond to the benzoic acid derivatives as numbered in Figure 3. Benzoic acid derivatives that were incorporated into a lincosamide are in bold and underlined.

Lincosamide library (Note for Figs. S6-S31)

Figures S6-S31 contain the structure, ion-extraceted LC-MS chromatograms, MS and collision-induced dissociation (CID) fragmentation MS spectra. The names of the lincosamides are derived by following these rules:

- The absence of methyl group is indicated by **OD** (*O*-demethyl), **ND** (*N*-demethyl), or **NDOD** (*N*-demethyl, *O*-demethyl).
- The benzoic acid derivative incorporated into the lincosamide is indicated by the number assigned to the derivative in Figure 3, e.g. CEL-2 contains the derivative **2**.
- **CEL** – compound derived from celesticetin (proline amino acid moiety); **CELIN** – compound derived from lincomycin (4-propyl-L-proline amino acid moiety).

Total number of possible combinations is 156; two compounds were not prepared (ODCEL-8, ODCEL-35) and four compounds (CEL, ODCEL, CEL-2, CEL-21) are not novel; 150 remaining lincosamides are novel.

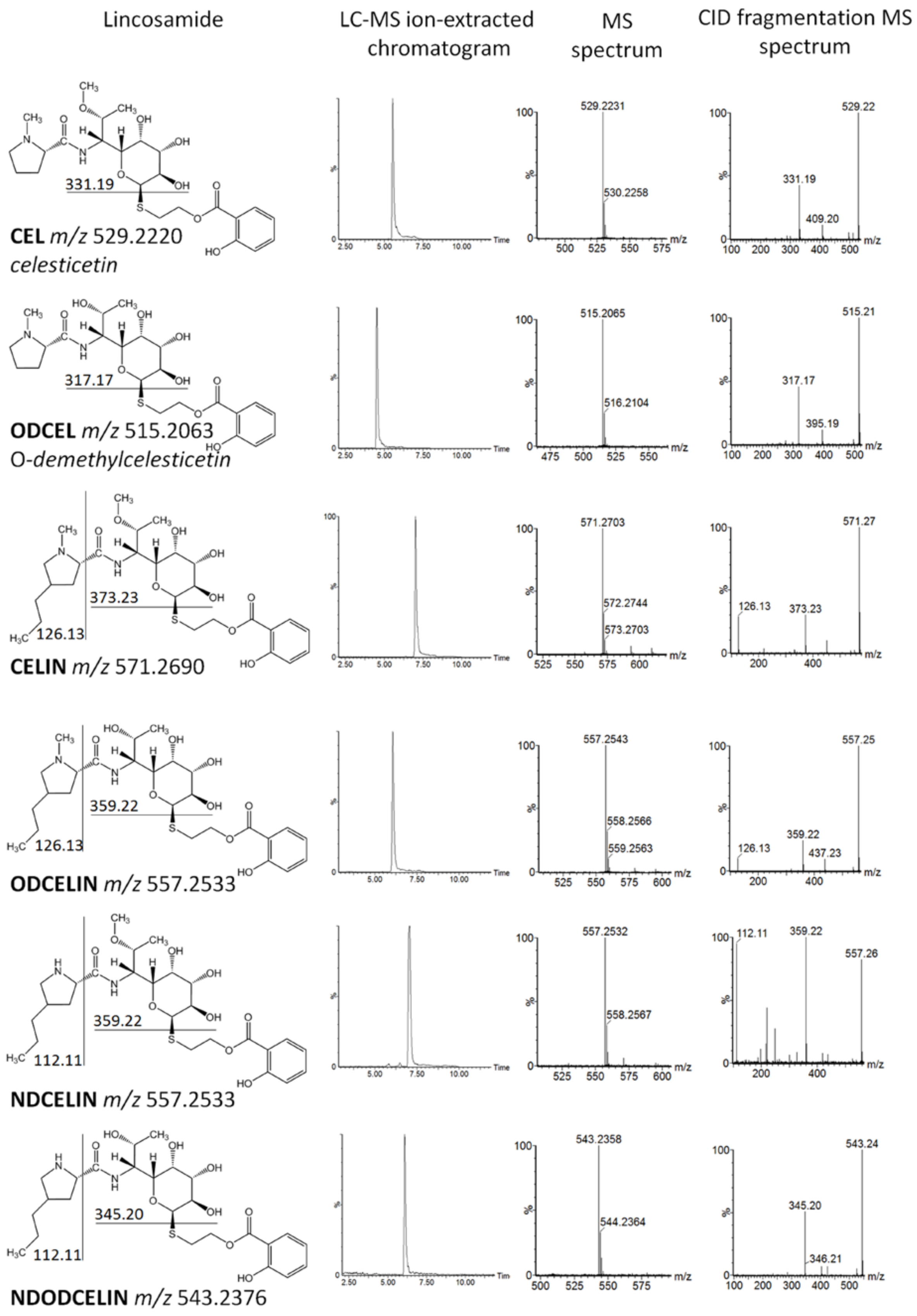

Fig. S6 Lincosamides with incorporated salicylic acid

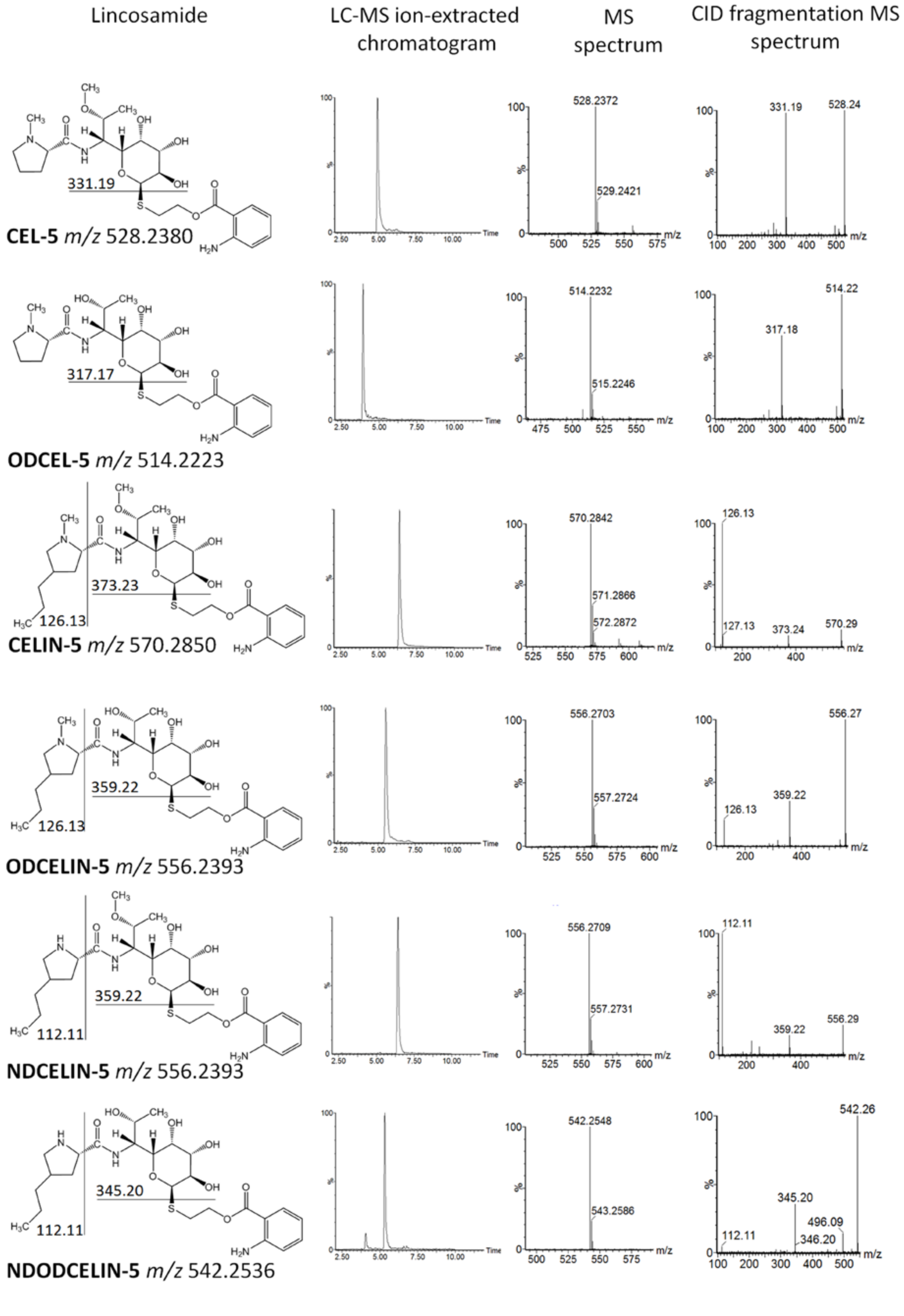

Fig. S7 Lincosamides with incorporated anthranilic acid

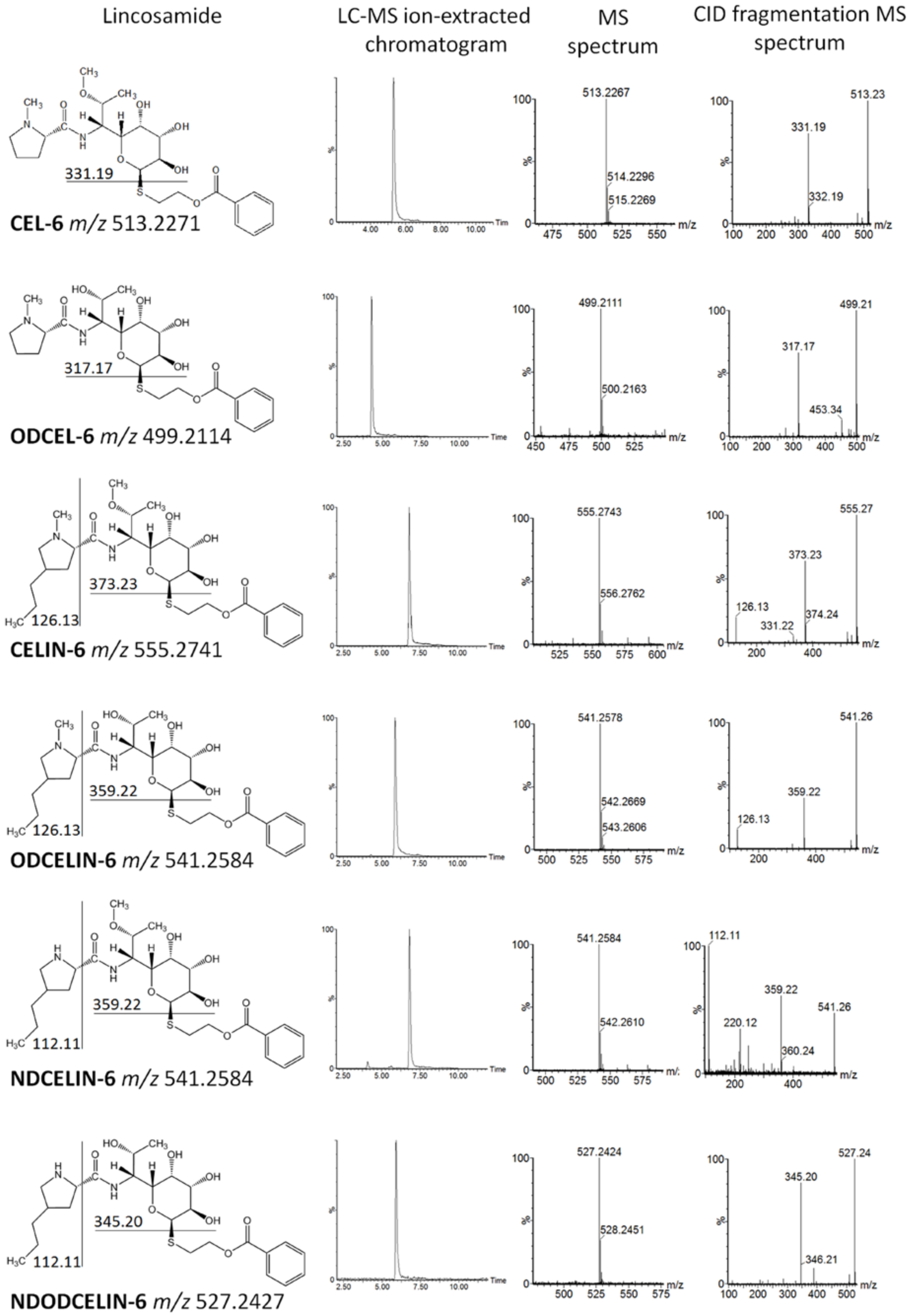

Fig. S8 Lincosamides with incorporated benzoic acid

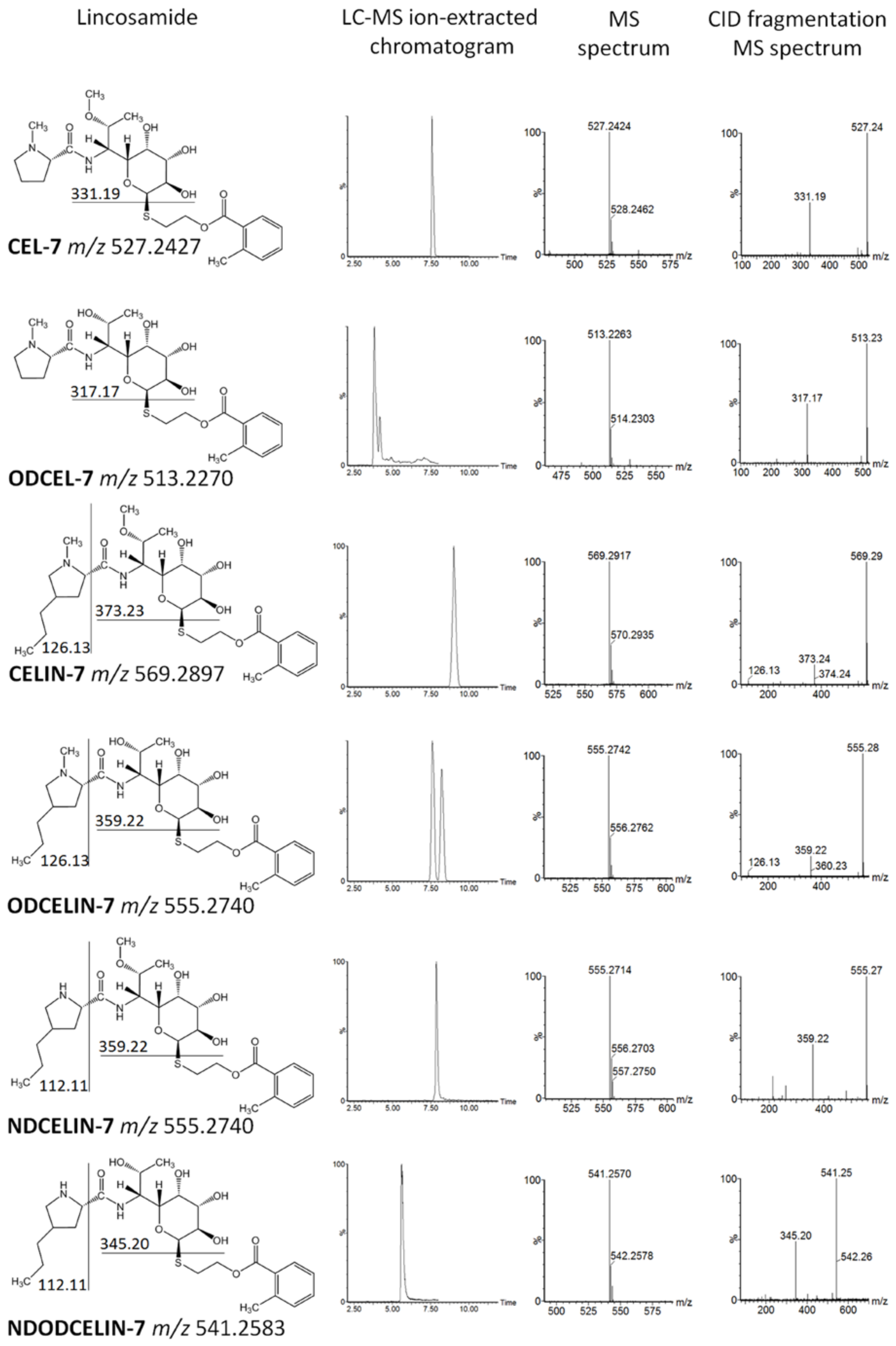

Fig. S9 Lincosamides with incorporated 2-methylbenzoic acid

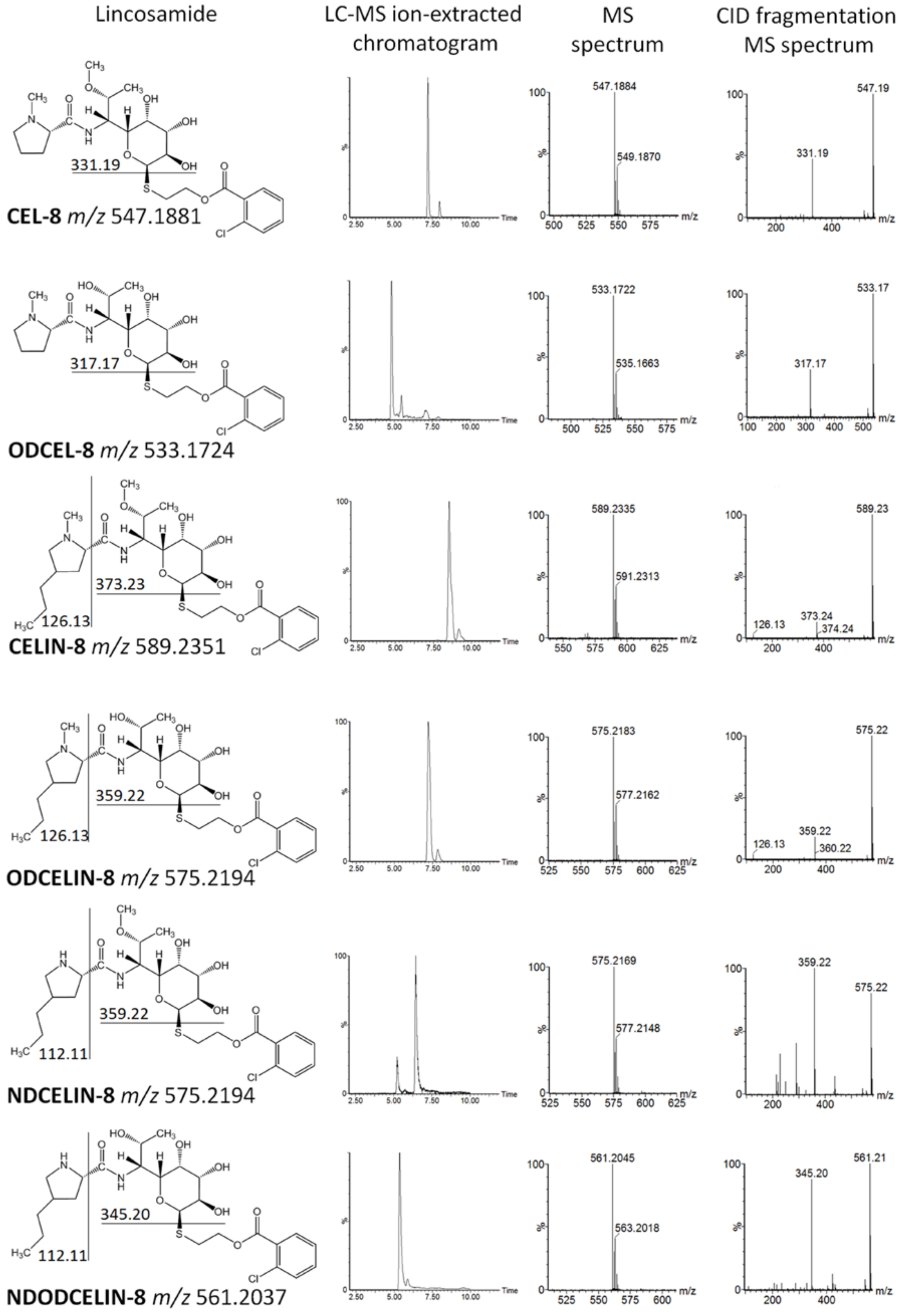

Fig. S10 Lincosamides with incorporated 2-chlorobenzoic acid

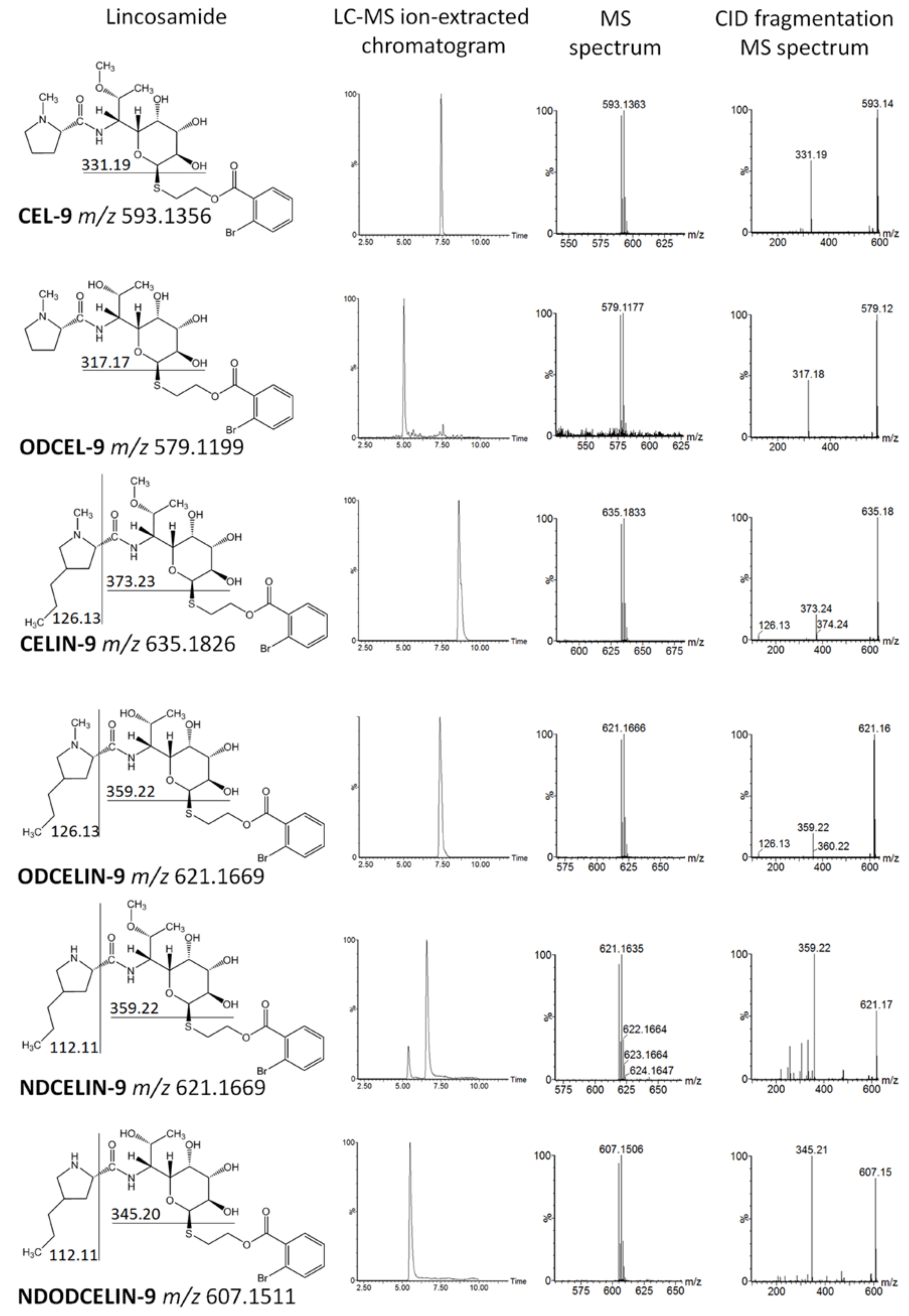

Fig. S11 Lincosamides with incorporated 2-bromobenzoic acid

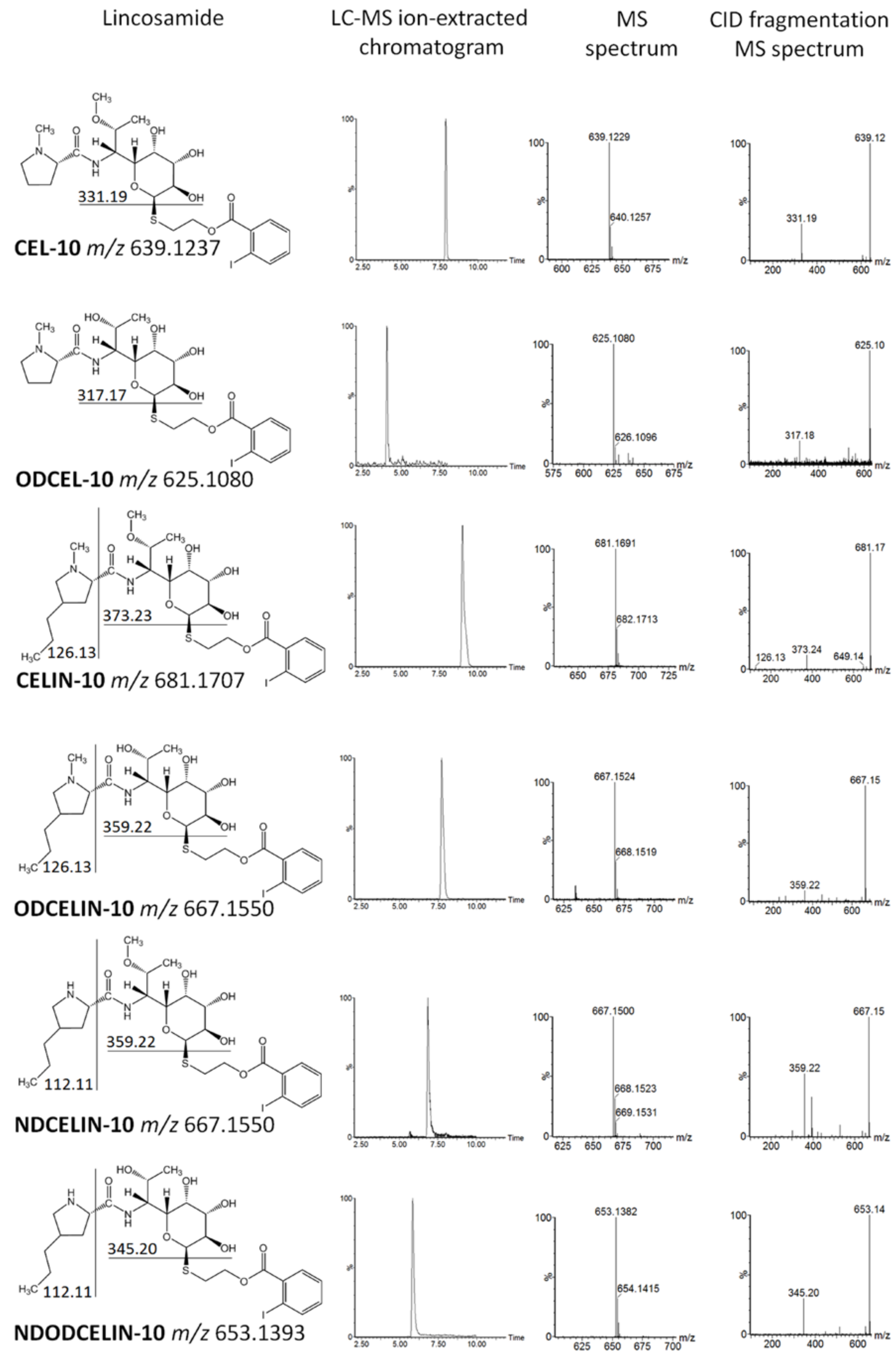

Fig. S12 Lincosamides with incorporated 2-iodobenzoic acid

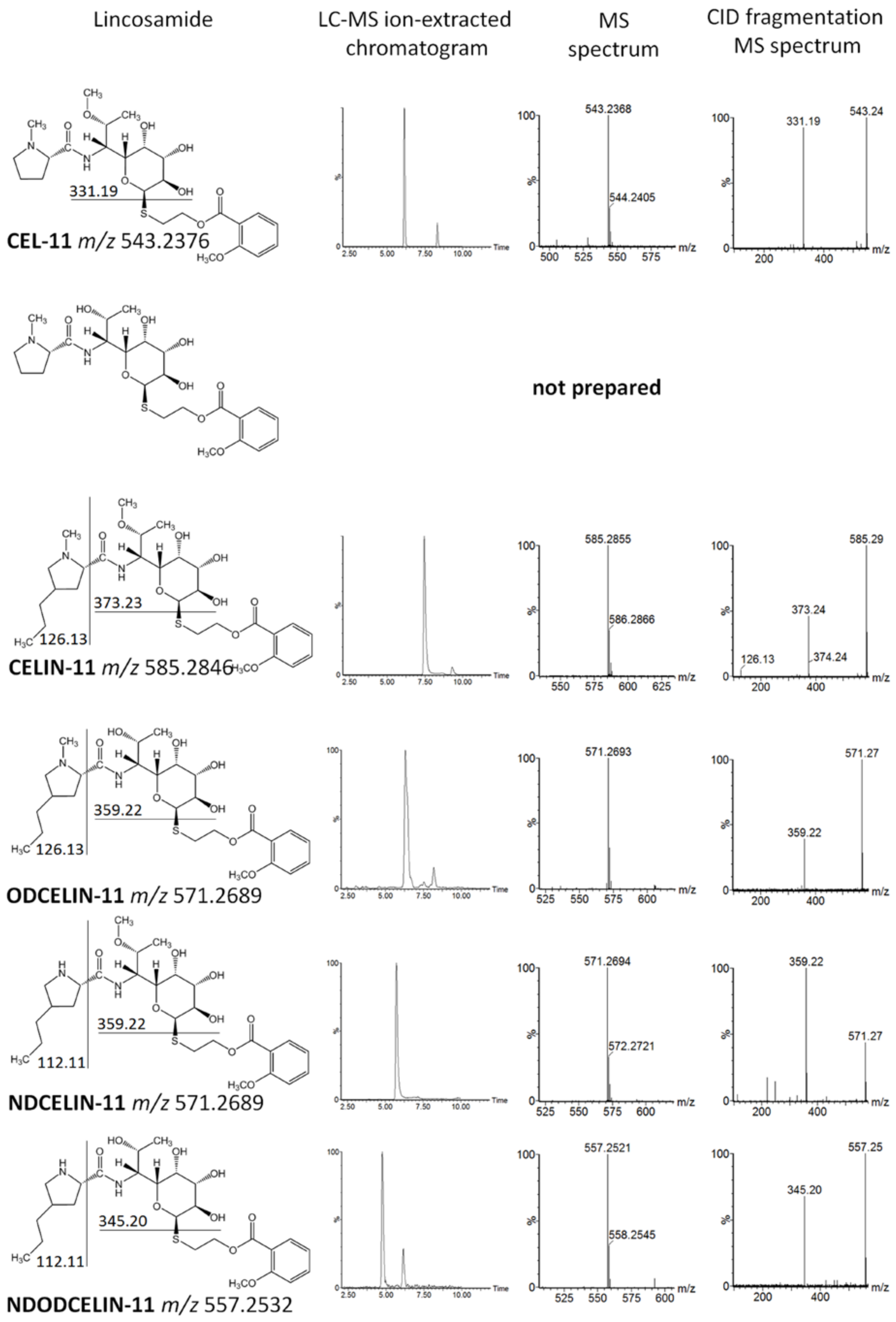

Fig. S13 Lincosamides with incorporated 2-methoxybenzoic acid

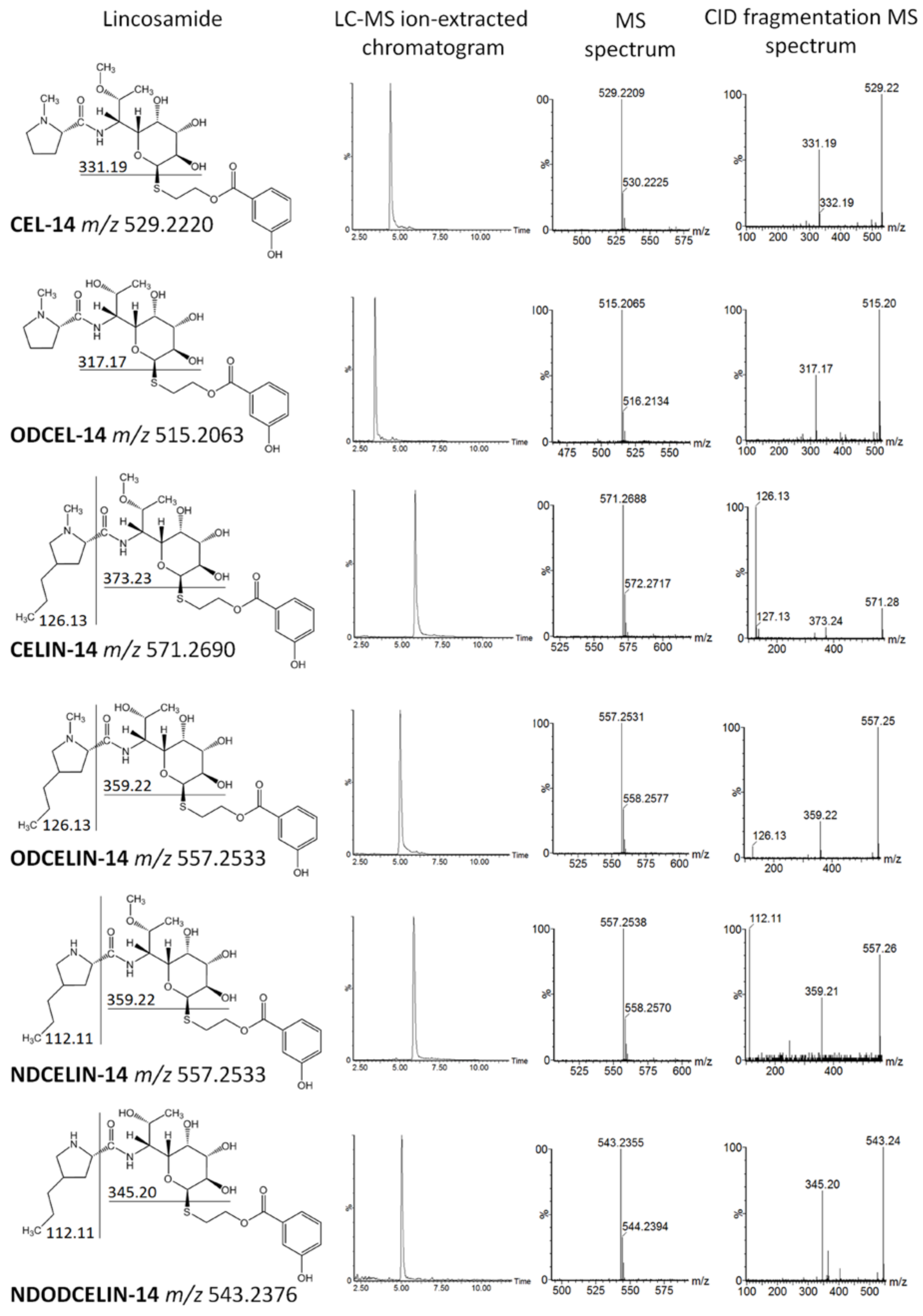

Fig. S14 Lincosamides with incorporated 3-hydroxybenzoic acid

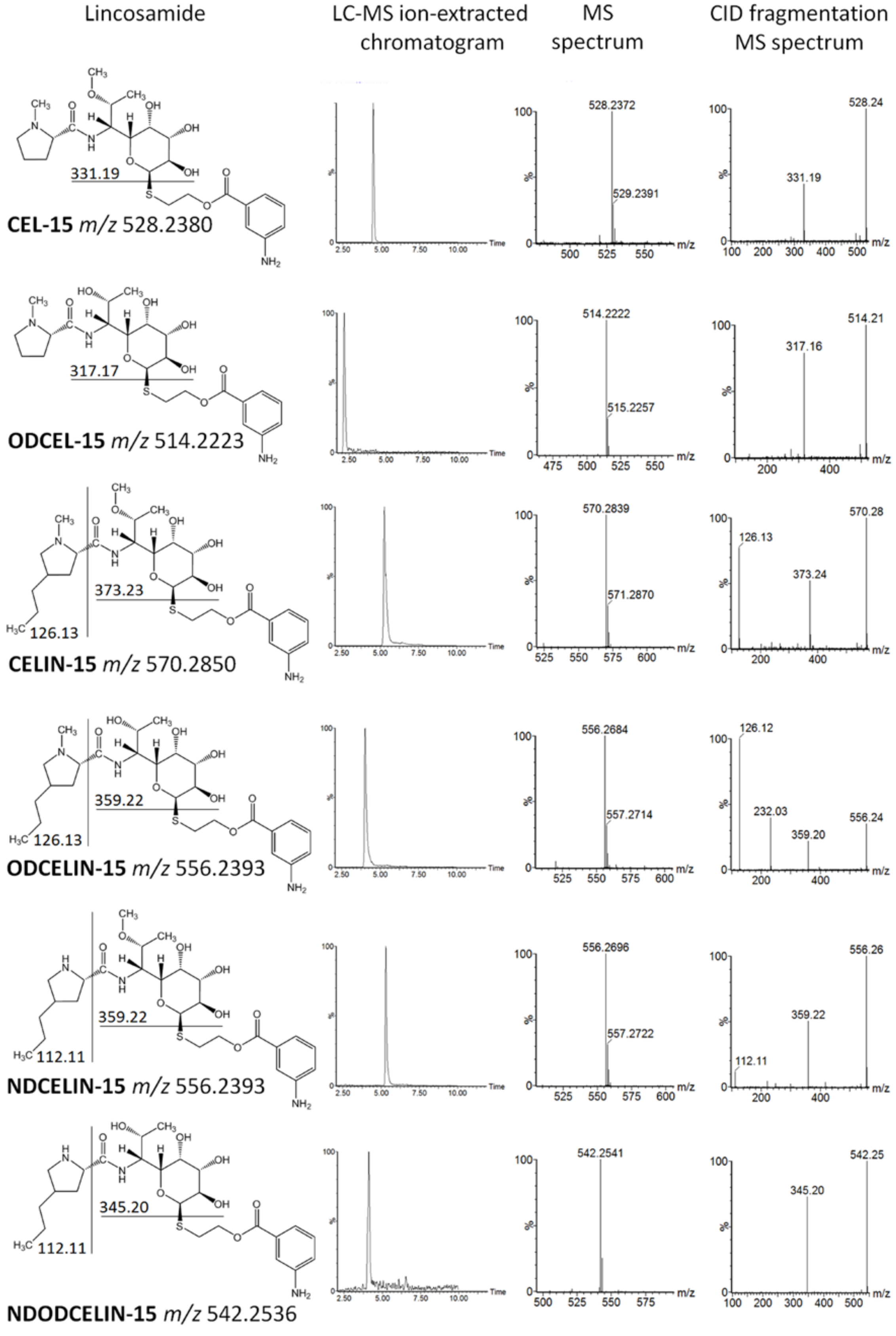

Fig. S15 Lincosamides with incorporated 3-aminobenzoic acid

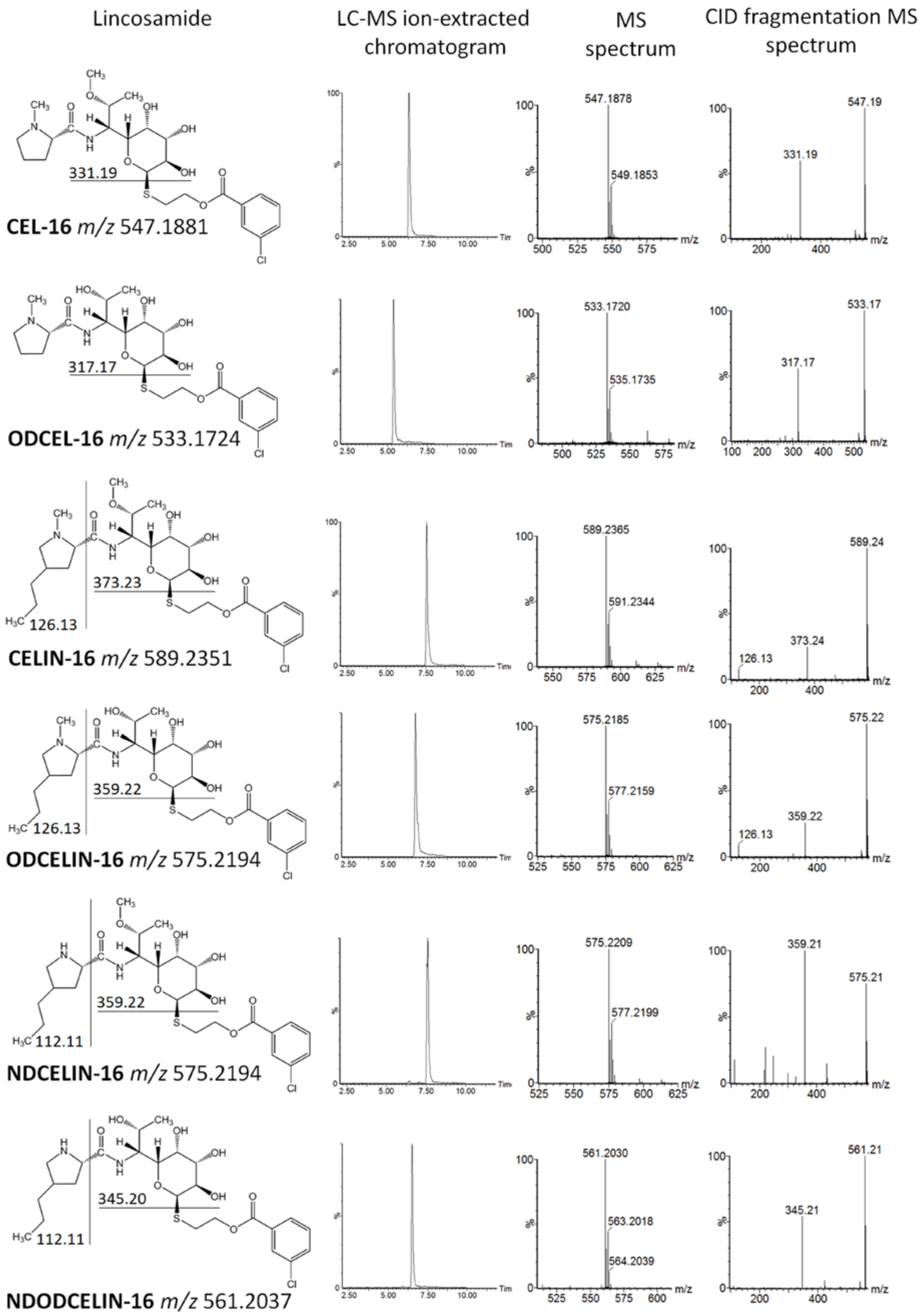

Fig. S16 Lincosamides with incorporated 3-chlorobenzoic acid

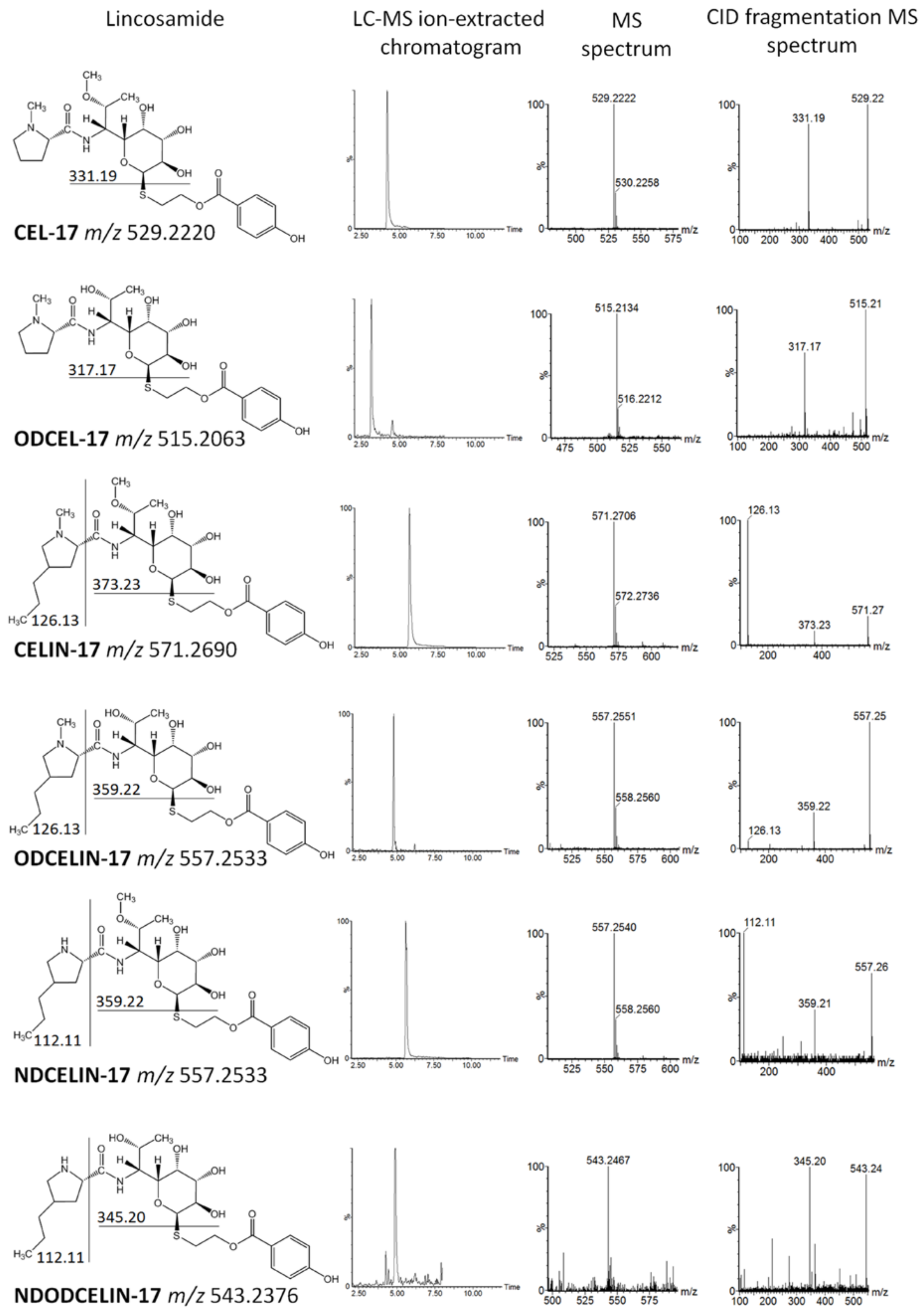

Fig. S17 Lincosamides with incorporated 4-hydroxybenzoic acid

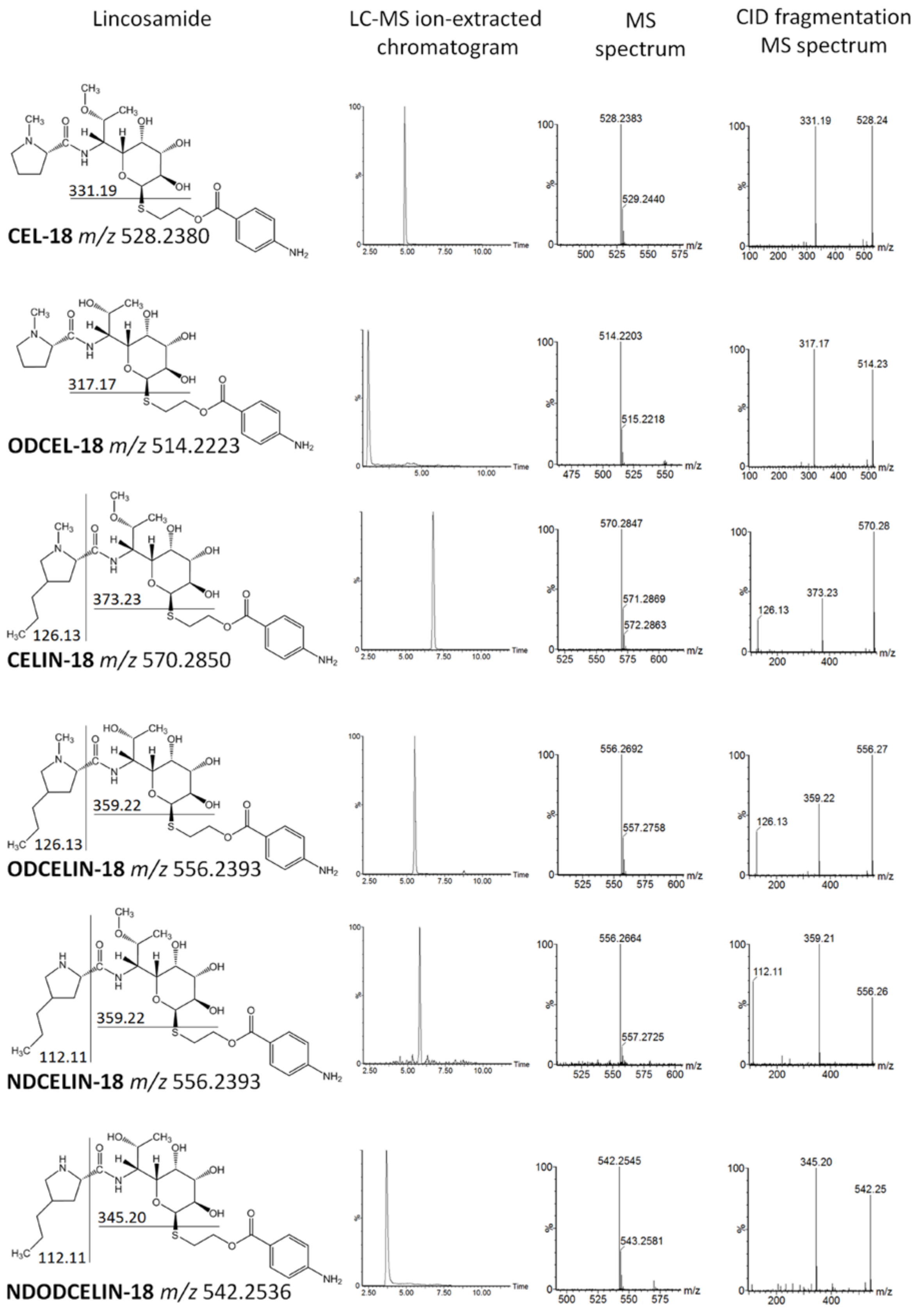

Fig. S18 Lincosamides with incorporated 4-aminobenzoic acid

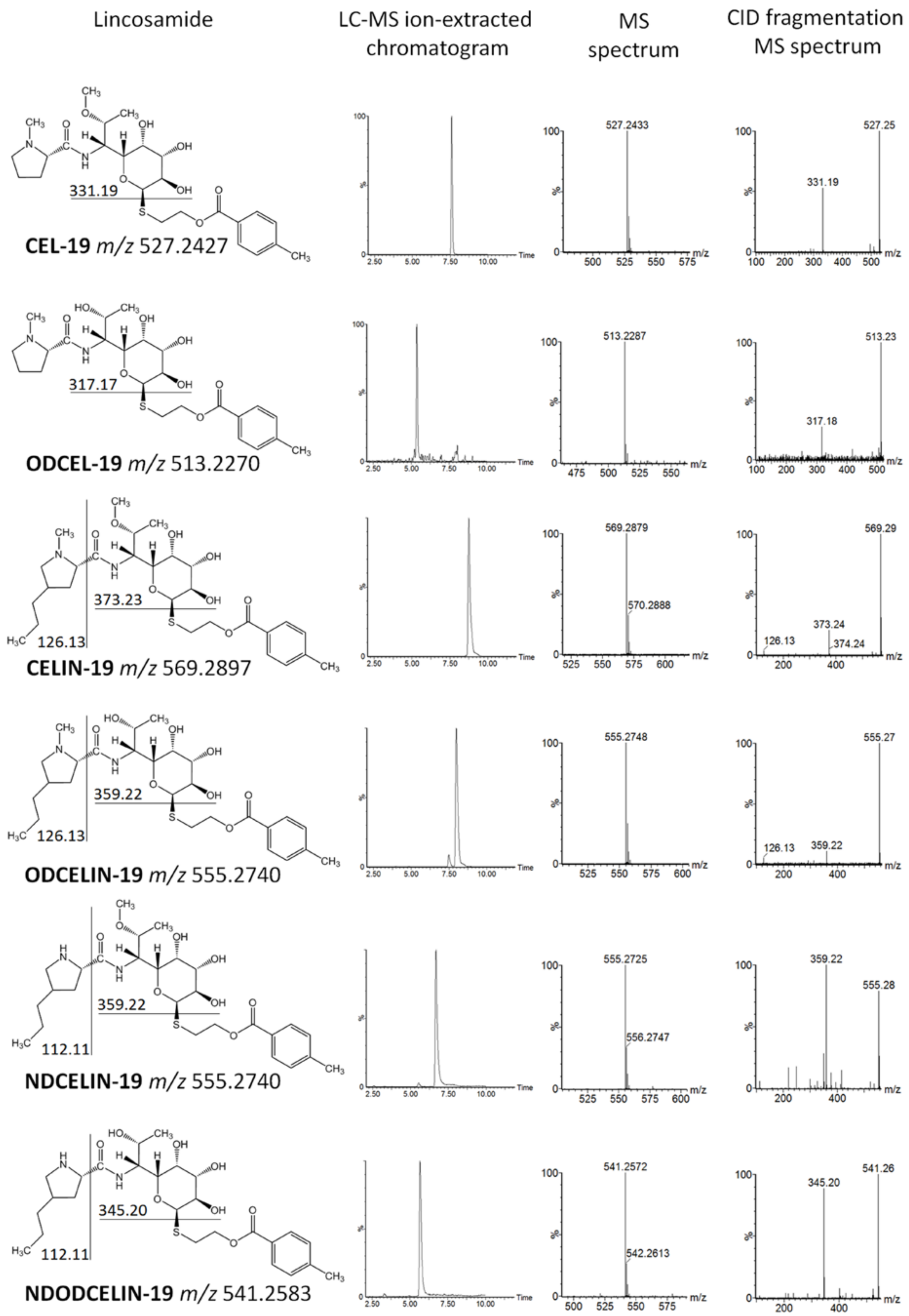

Fig. S19 Lincosamides with incorporated 4-methylbenzoic acid

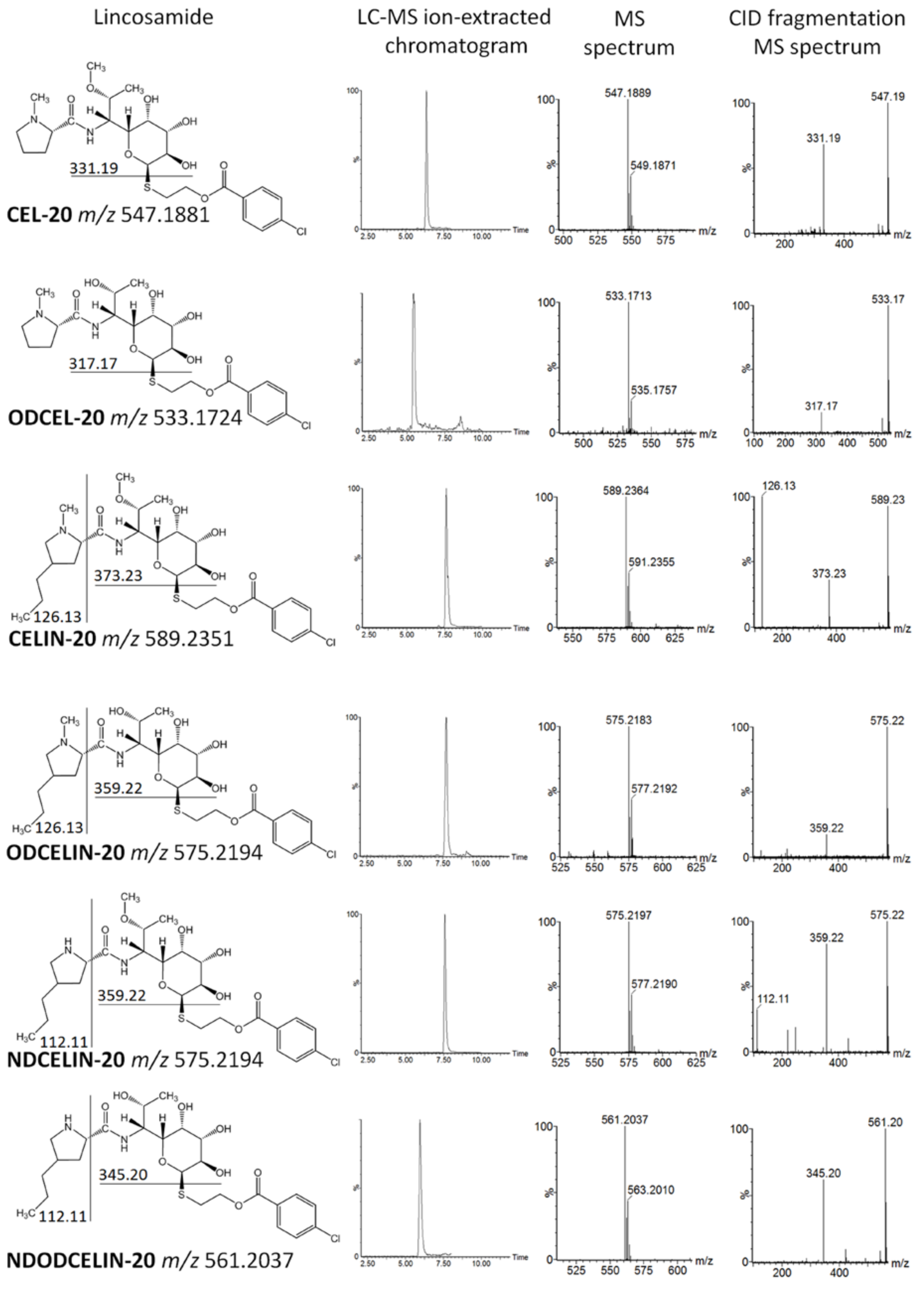

Fig. S20 Lincosamides with incorporated 4-chlorobenzoic acid

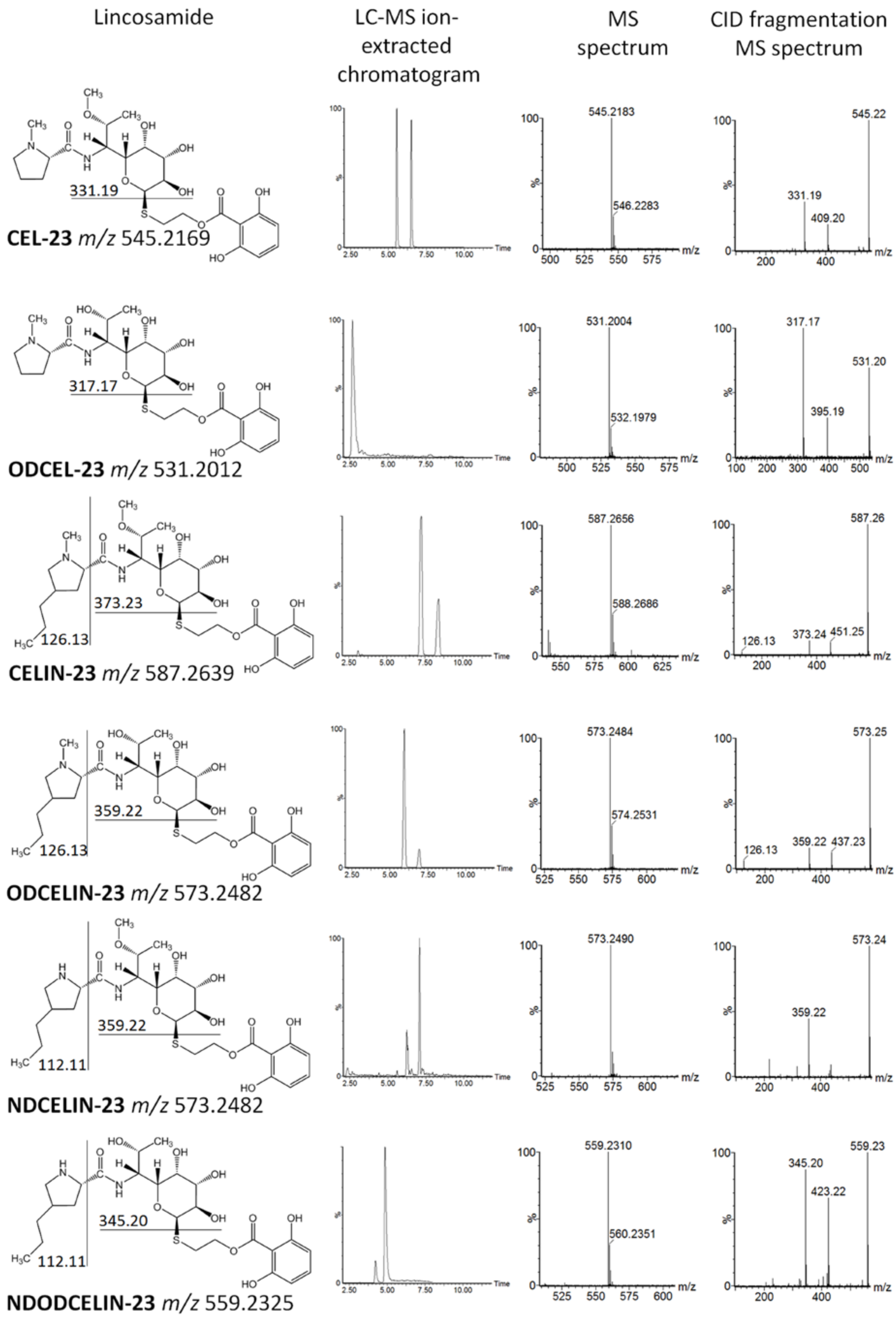

Fig. S21 Lincosamides with incorporated 2,6-dihydroxybenzoic acid

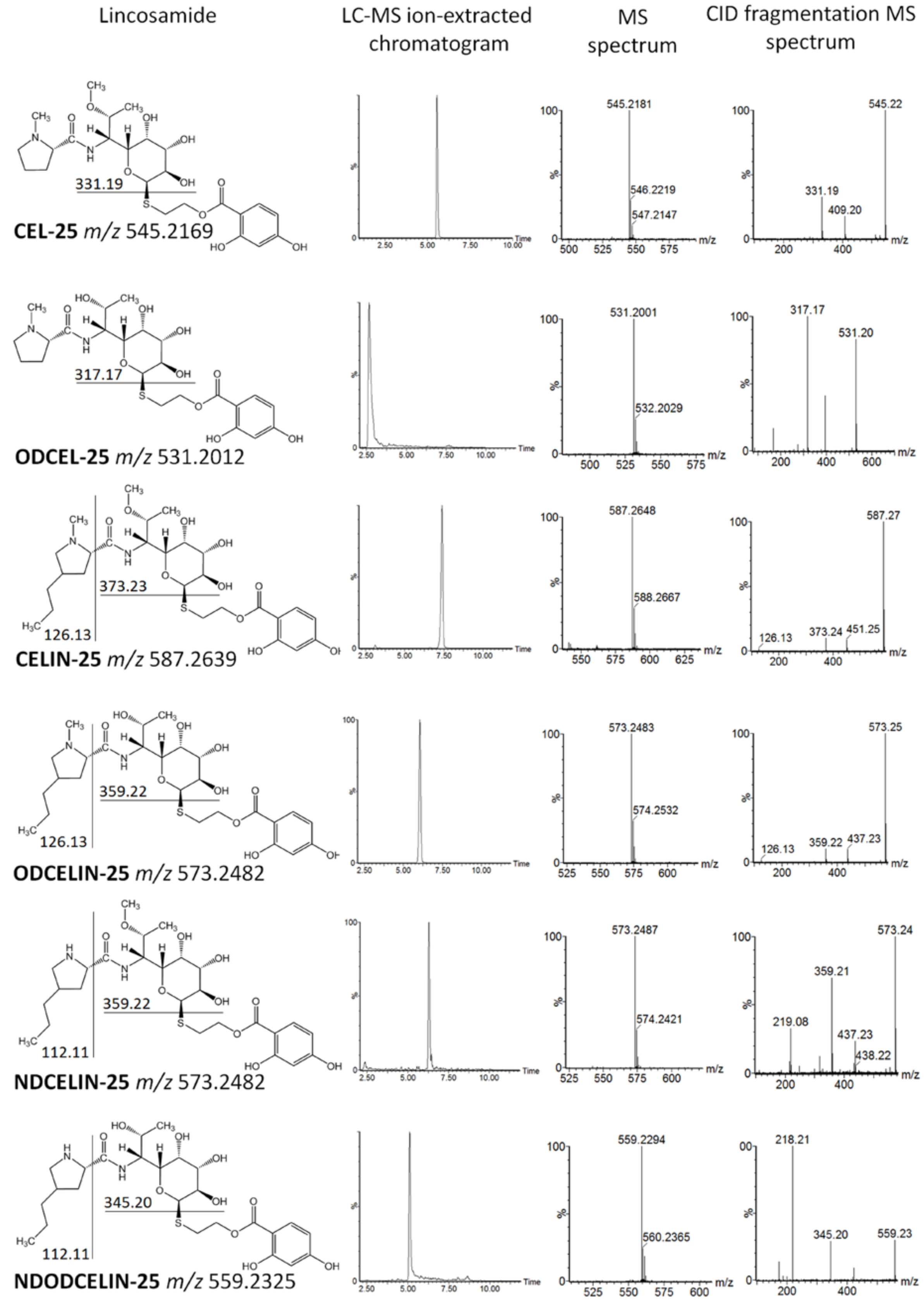

Fig. S22 Lincosamides with incorporated 2,4-dihydroxybenzoic acid

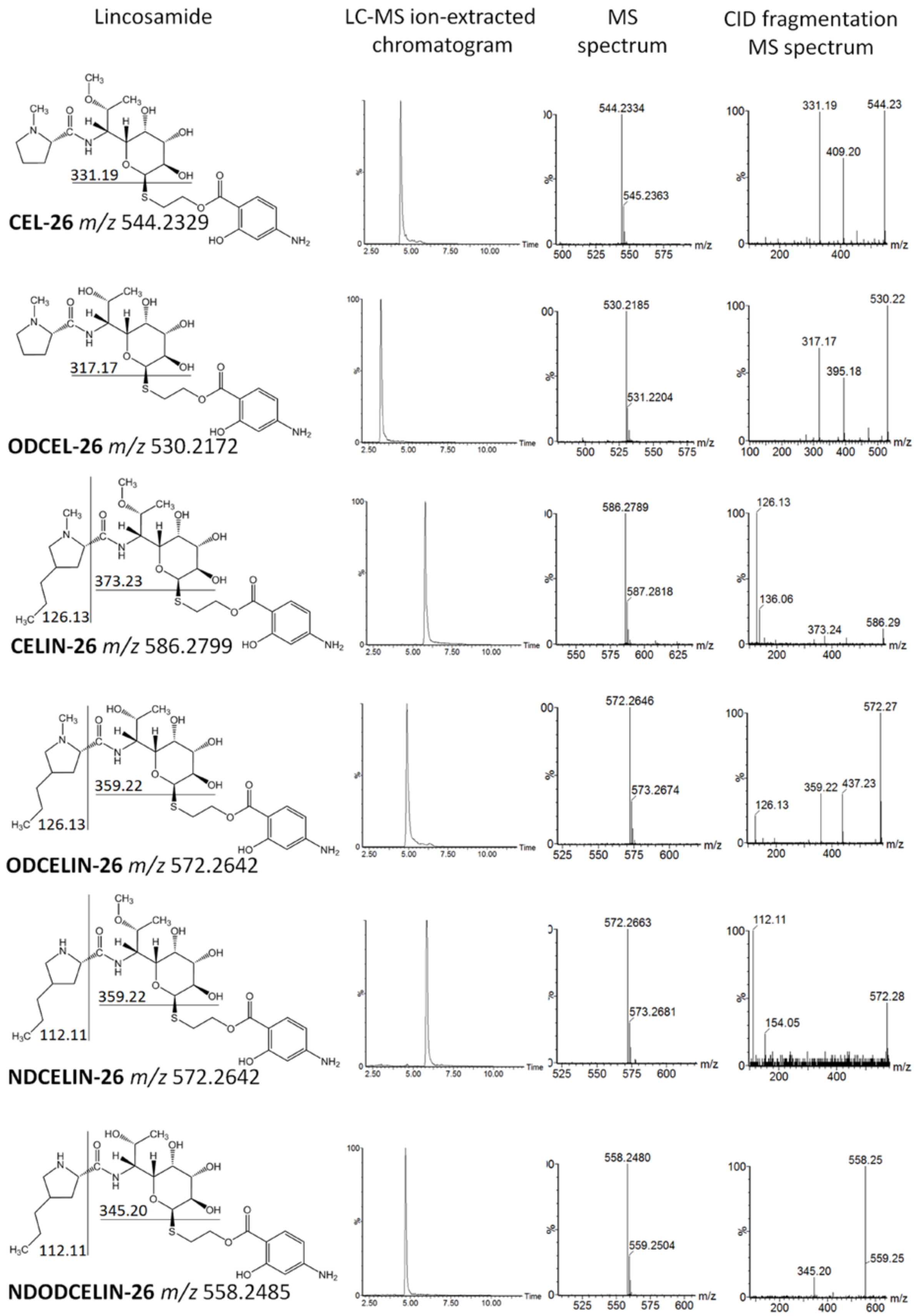

Fig. S23 Lincosamides with incorporated 4-amino-2-hydroxybenzoic acid

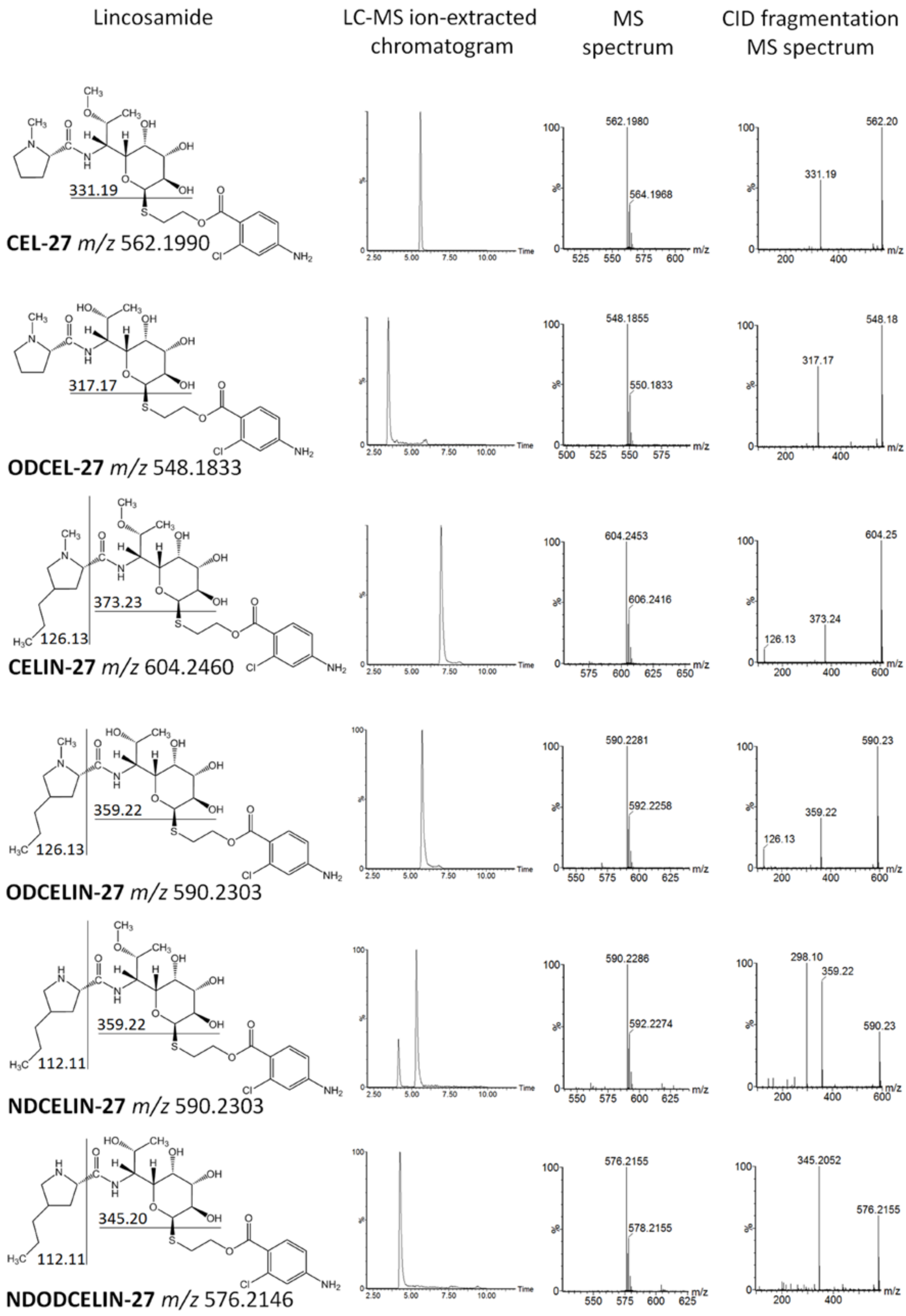

Fig. S24 Lincosamides with incorporated 4-amino-2-chlorobenzoic acid

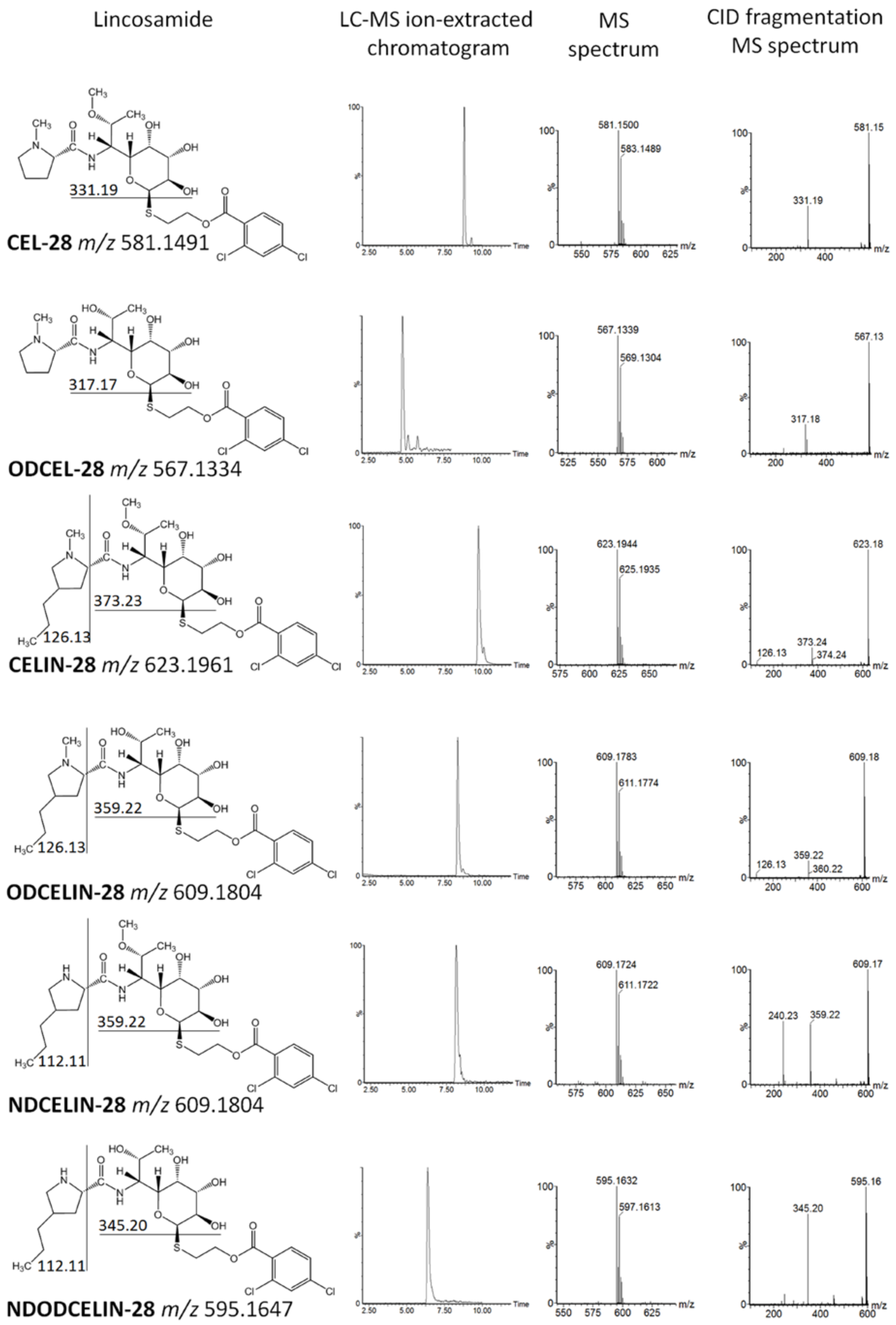

Fig. S25 Lincosamides with incorporated 2,4-dichlorobenzoic acid

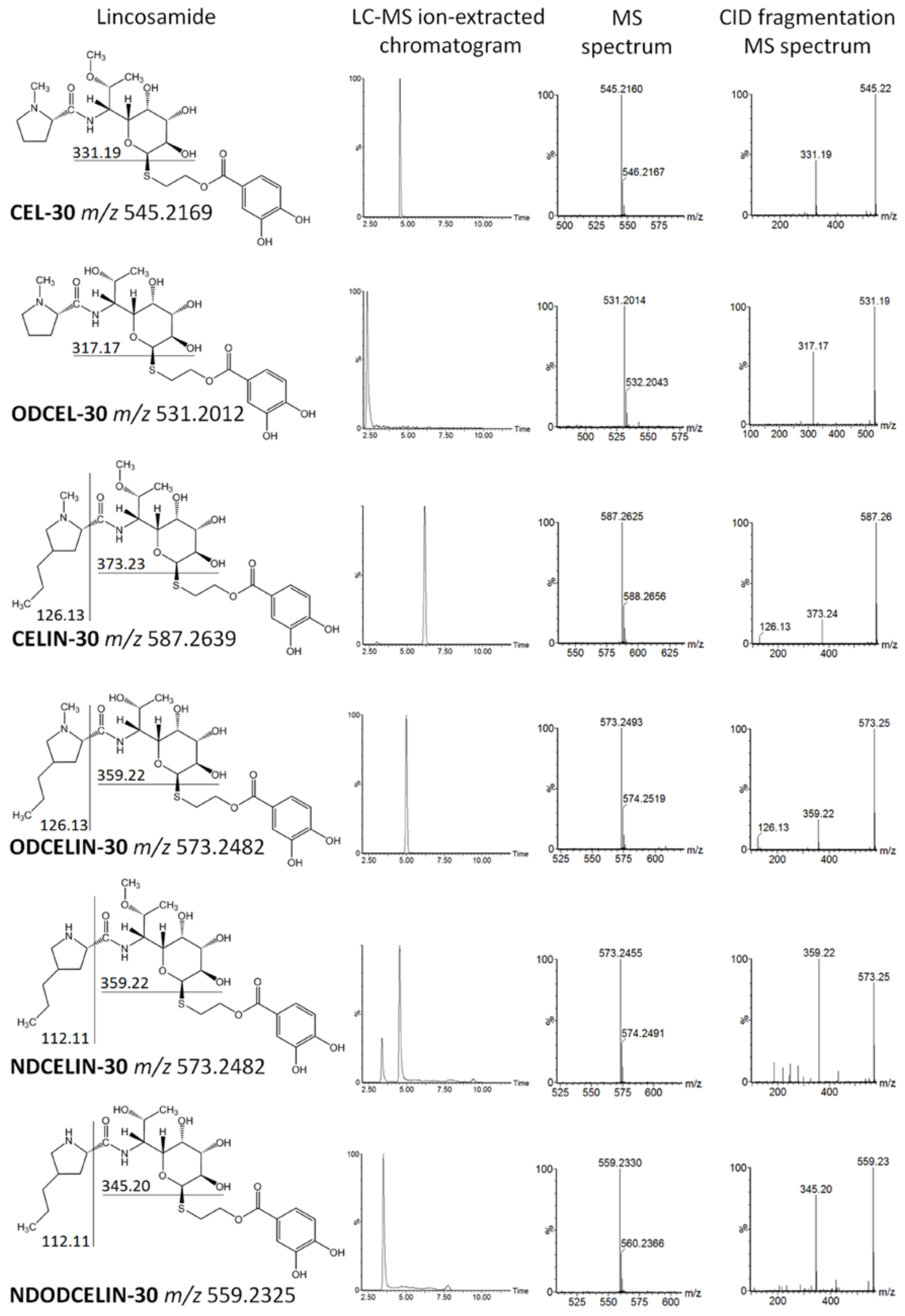

Fig. S26 Lincosamides with incorporated 3,4-dihydroxybenzoic acid

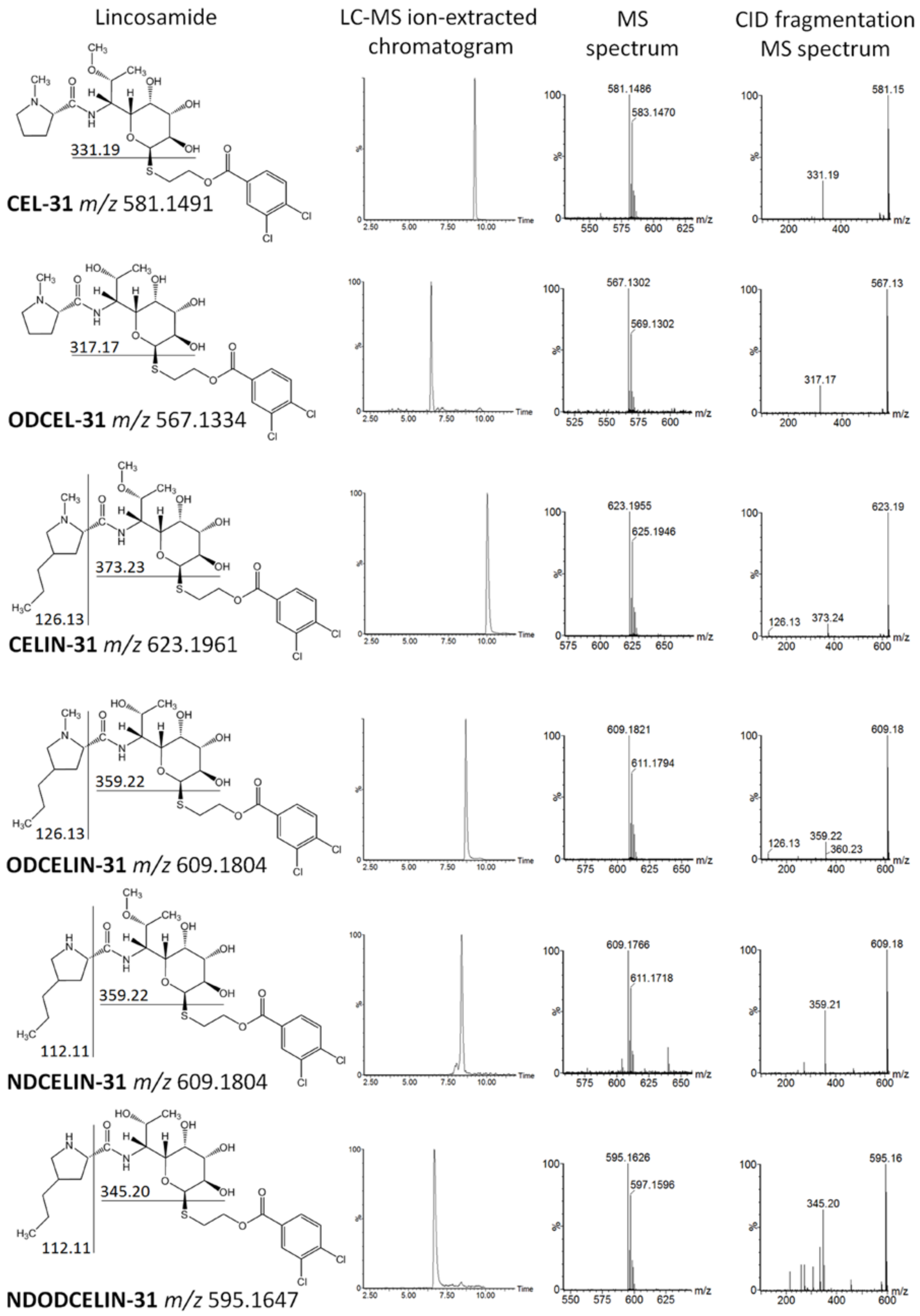

Fig. S27 Lincosamides with incorporated 3,4-dichlorobenzoic acid

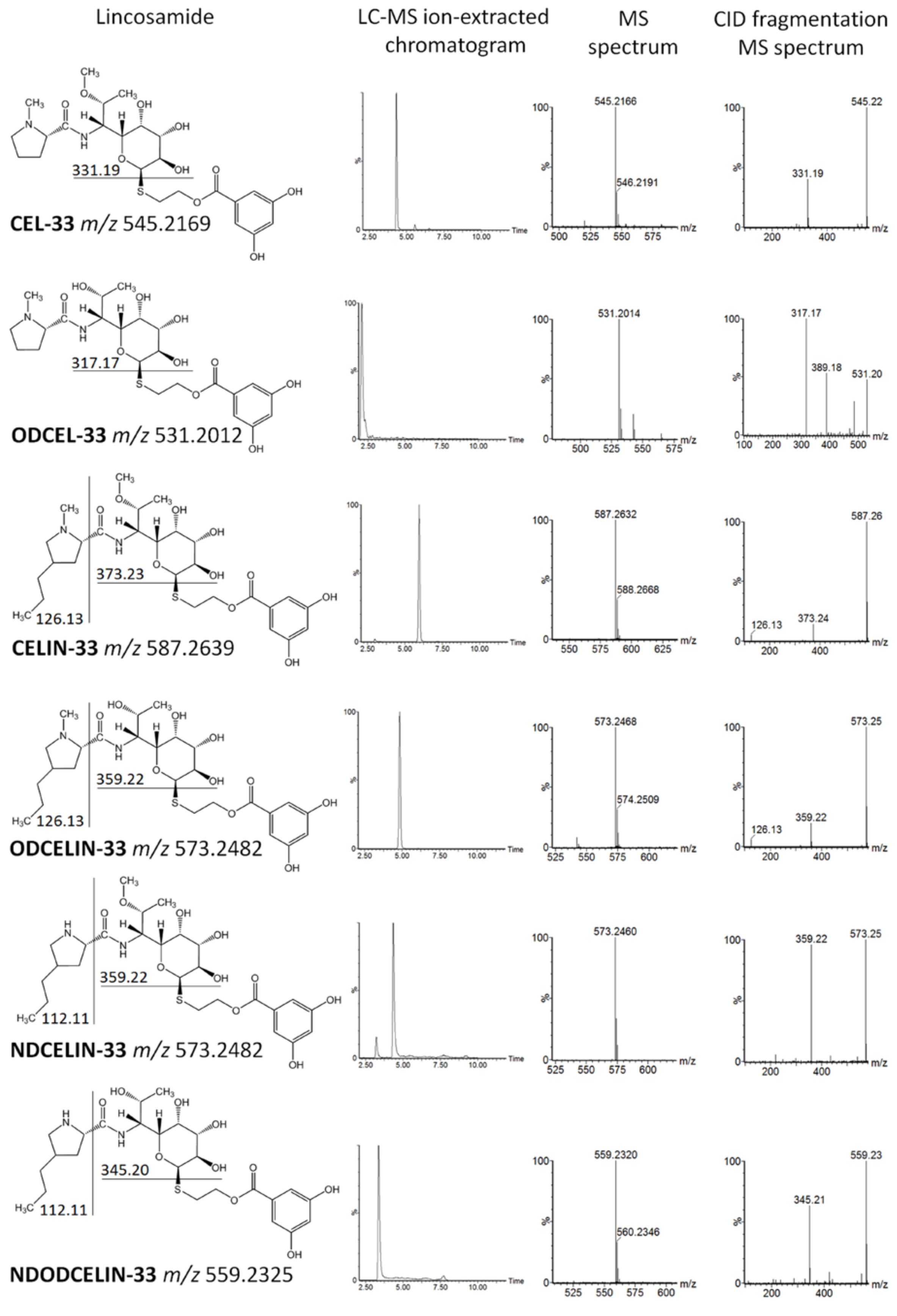

Fig. S28 Lincosamides with incorporated 3,5-dihydroxybenzoic acid

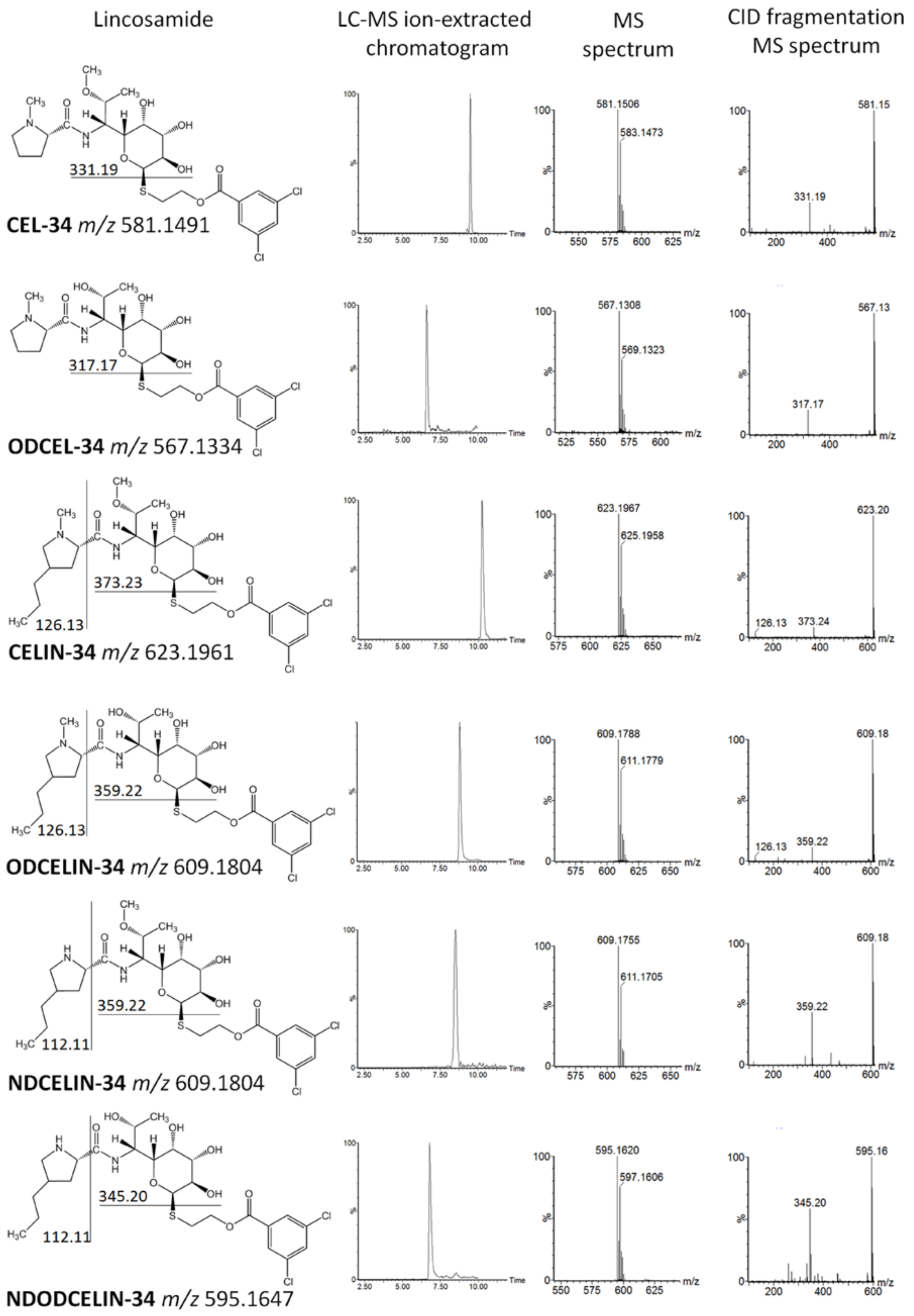

Fig. S29 Lincosamides with incorporated 3,5-dichlorobenzoic acid

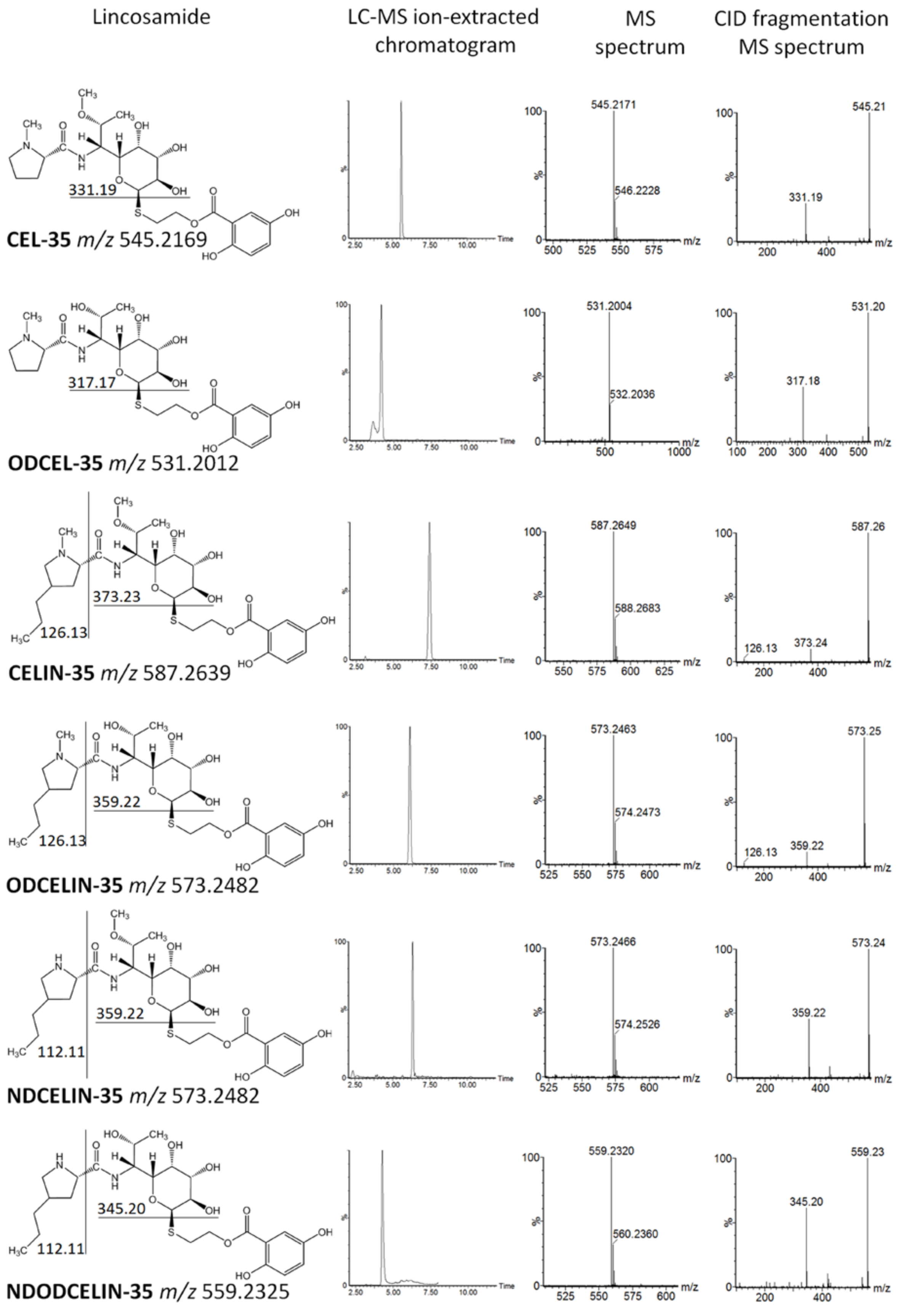

Fig. S30 Lincosamides with incorporated 2,5-dihydroxybenzoic acid

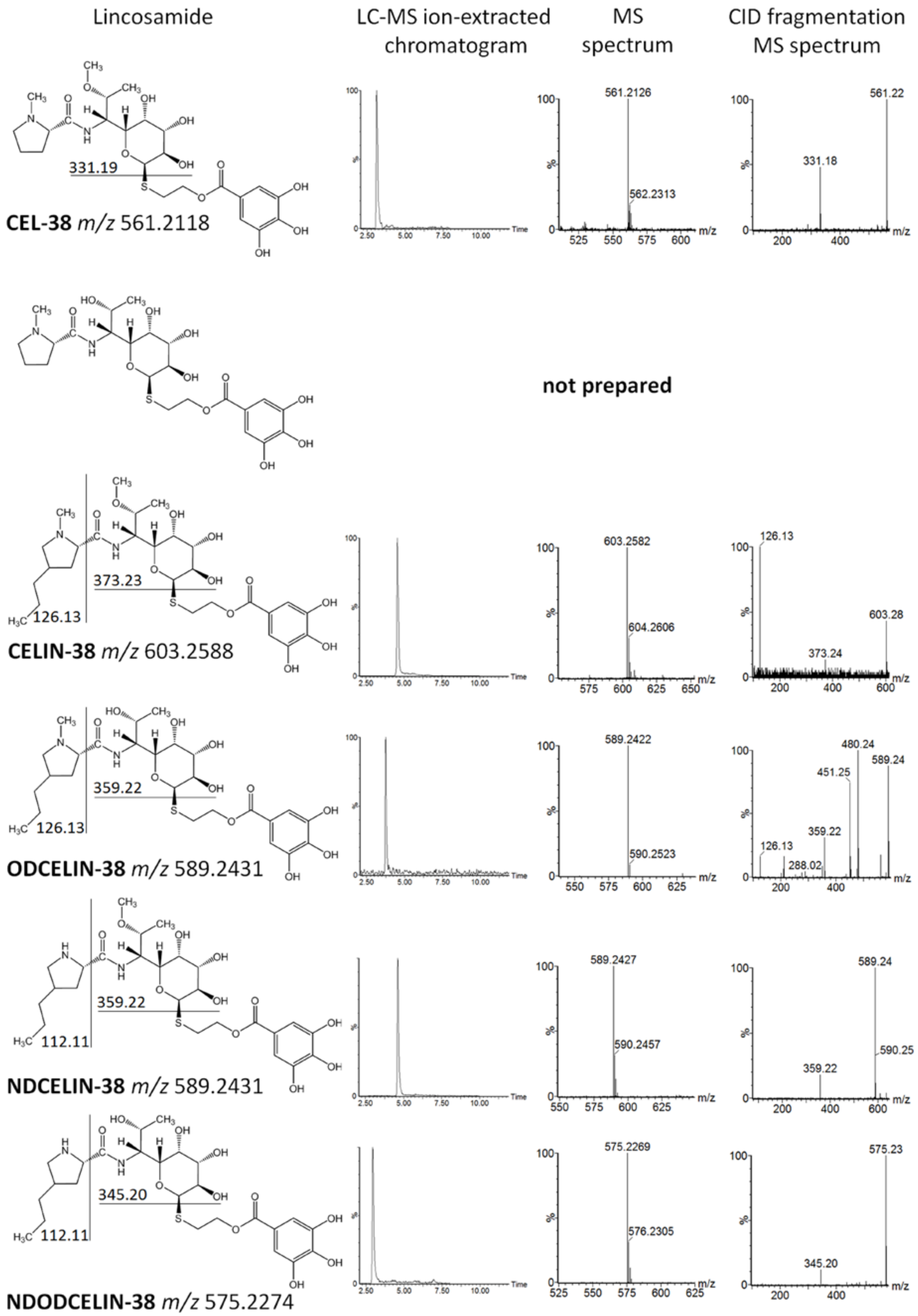

Fig. S31 Lincosamides with incorporated 3,4,5-trihydroxybenzoic acid

Supplementary references

1 J. Janata, S. Kadlcik, M. Koberska, D. Ulanova, Z. Kamenik, P. Novak, J. Kopecky, J. Novotna, B. Radojevic, K. Plhackova, R. Gazak, L. Najmanova, *PLoS One*, 2015, **10**, e0118850.

2 T. Kieser, M. Bibb, M. Buttner, K. Chater, D. Hopwood, Practical Streptomyces Genetics. Norwich, UK: The John Innes Foundation. 2000, 613.

3 L. Najmanova, D. Ulanova, M. Jelinkova, Z. Kamenik, E. Kettnerova, M. Koberska, R. Gazak, B. Radojevic, J. Janata, *Folia Microbiol*., 2014, **59**, 543.

4 R. M. Atlas, Handbook of microbiological media; R. M. Atlas, editor: CRC press. 2010.

5 H. Schagger, W. A. Cramer, G. von Jagow, *Anal. Biochem.*, 1994, **217**, 220.

6 M. Kearse, R. Moir, A. Wilson, S. Stones-Havas, M. Cheung, S. Sturrock, S. Buxton, A. Cooper, S. Markowitz, C. Duran, T. Thierer, B. Ashton, P. Mentjies, A. Drummond, *Bioinformatics*, 2012, **28**, 1647.

7 K. Katoh, D. M. Standley, *Mol. Biol. Evol.*, 2013, **30**, 772.

8 European Committee for Antimicrobial Susceptibility Testing (EUCAST), Clin Microbiol Infect, 2003, **9** (8), ix-xv.
